# A Tutorial Review on Surface Plasmon Resonance Biosensors: Applications in Biomedicine

**DOI:** 10.1021/acsbiomedchemau.5c00182

**Published:** 2025-11-23

**Authors:** Antony Chirco, Elisabetta Meacci, Giancarlo Margheri

**Affiliations:** † Department of Experimental and Clinical Biomedical Sciences “Mario Serio”, 9300 University of Florence, 50134 Firenze, Italy; ‡ Institute for Complex Systems of the National Council of Researchers of Italy, 50019 Sesto Fiorentino, Florence, Italy

**Keywords:** surface plasmon resonance, biosensors, biomedicine, diagnostics, biomarker detection, machine learning, point of care, biosensing techniques

## Abstract

Surface Plasmon Resonance (SPR) has proven to be one of the most effective technologies in terms of specificity, affinity, and determination of kinetic parameters for evaluating interactions between macromolecules. The focus of this tutorial is to give an overview of the recent advances and applications of SPR biosensors in biomedicine that are presented emphasizing the potentiality for the detection of very low abundant compounds, which, in recent years, have assumed great importance for prevention and early diagnosis of various diseases in biomedicine. The real-time detection of important biomarkers such as tumor markers, viruses, and toxins but also of compounds of interest such as drugs and hormones allows point-of-care analysis and monitoring of disease progression quickly and in a less invasive manner. Over the past years, several technical innovations have been introduced to SPR devices, which have gone through a process of miniaturization, portability, flexibility, and cost reduction. These characteristics are in line with the advantages of SPR biosensors over other biosensing techniques, i.e., to be label-free detection systems and their capacity to observe in real-time the interactions between a variety of molecules of interest at the metal surface. Recent advances in SPR sensor technology, such as LSPR, SPRi, and SPRM, attempted to improve the sensitivity and performance of molecule detection.

## Graphical abstract

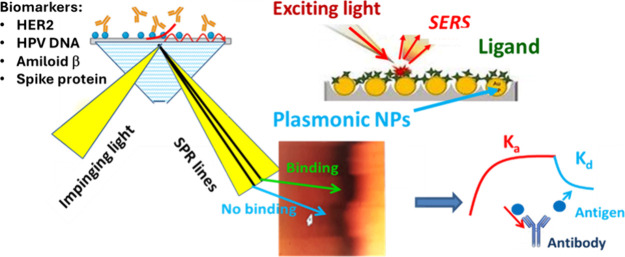

## 1 Introduction

Since its introduction in the 1980s, SPR has emerged as one of the most powerful label-free analytical techniques for studying macromolecular interactions, offering exceptional specificity, sensitivity, and the ability to determine kinetic parameters.^1^ This optical sensing method detects subtle variations in the refractive index that occur near the surface of thin metallic films, typically composed of gold, silver, aluminum, or similar materials, when biomolecular interactions take place. The principle of SPR is rooted in the phenomenon of Attenuated Total Reflection (ATR). Under this condition, the free electrons in a noble metal resonate collectively with an incident electromagnetic field, producing a characteristic decrease in reflectivity at a specific incidence angle known as the resonance angle. This angle varies depending on the wavelength of the incident light and the optical properties of the surrounding medium. In an SPR experiment, biorecognition elements, such as antibodies, enzymes, peptides, or DNA strands, are immobilized onto the metallic surface of the sensor chip. When a solution containing the target analyte flows across the surface, binding interactions between the analyte and immobilized receptors induce a change in the local refractive index. This results in a shift of the resonance angle, which can be detected by monitoring the reflected light intensity as a function of the angle of incidence during the receptor–ligand binding process (Figure 1).

**Figure 1:**
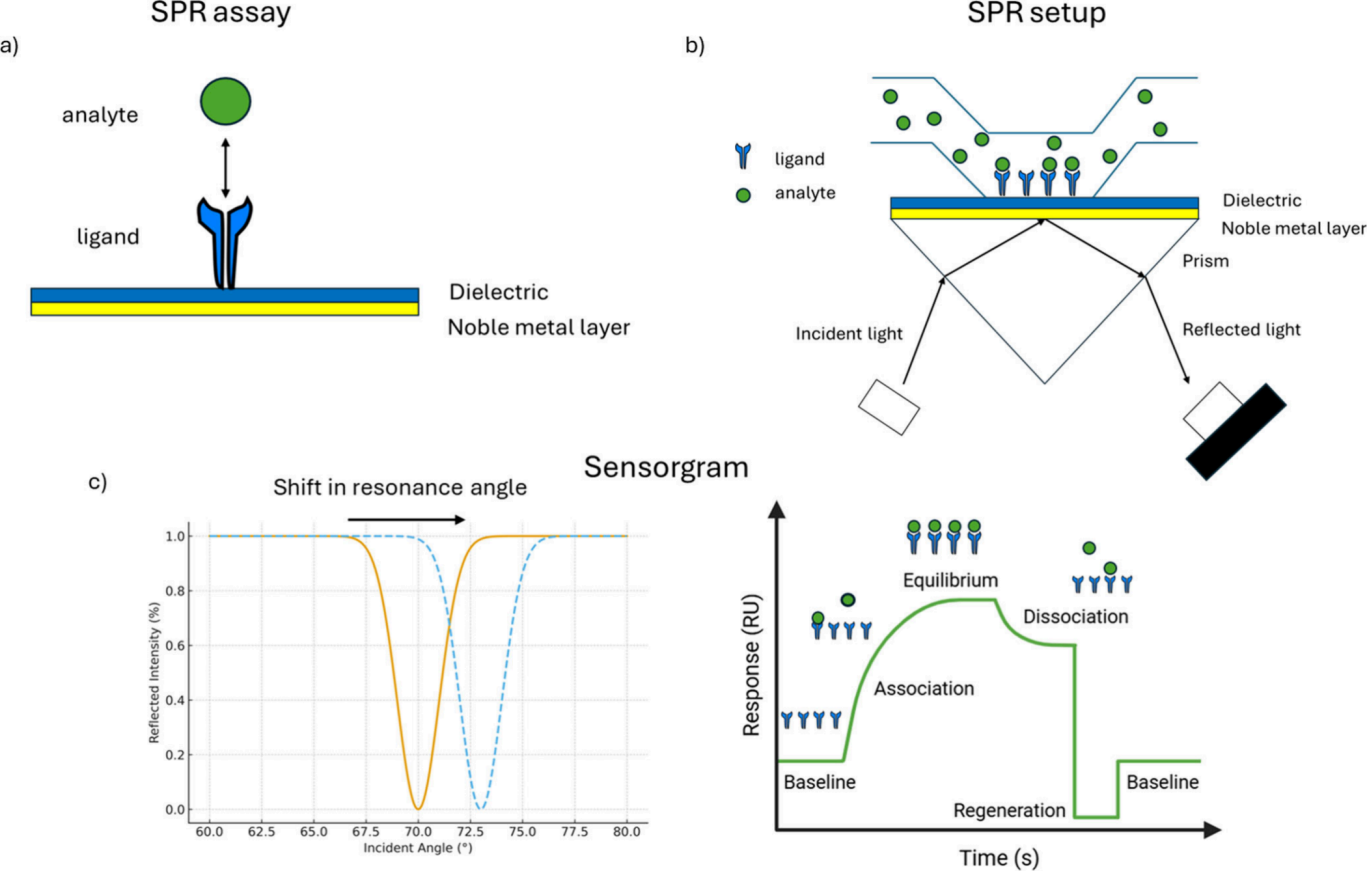
Schematic representation of the SPR setup. a) In a standard SPR assay, one molecule, termed the ligand, is immobilized on the sensor surface. The sensor is prefunctionalized with specific surface chemistries to facilitate ligand attachment, enabling optimal interaction with its binding partner, the analyte. b) A solution containing the analyte is then passed over the functionalized sensor surface, where ligand–analyte binding occurs through specific molecular recognition. c) The minimum in reflected light intensity shifts as the angle of incidence changes, corresponding to variations in the refractive index caused by mass accumulation on the sensor surface. This shift is recorded in a sensorgram that depicts the real-time association and dissociation kinetics of the analyte–ligand interaction as a function of time.

Numerous studies have encouraged the potential of SPR biosensors by increasing the effectiveness of the technique and by monitoring biochemical and chemical species interactions through the basic theory of SPR sensing.

## 2 Applications

### 2.1 Biomedicine

The applications of SPR biosensors in the biomedical field are countless, and a large variety of biomolecules can be studied.^1^

Certainly, SPR biosensors are used to study the interactions between bioactive molecules based on the analysis of affinity binding of a broad spectrum of bonds: the antibody–antigen binding,^2,3^ kinetics between ligand and receptor,^4,5^ the reaction enzyme–substrate^6,7^ and epitope mapping.^8,9^ The advent of click chemistry has allowed the scientific community to design nucleic acid analogues with innovative properties and improved stability, functionality and binding aspects that can be used to develop new therapeutic agents or diagnostic tools.^10^ SPR enables quantitative characterization of biomolecular interactions, providing key parameters such as binding affinity, association, and dissociation rate constants. Through kinetic and equilibrium analyses, SPR allows the elucidation of molecular recognition mechanisms, distinguishing between high and low-affinity interactions, transient versus stable complexes, and single-step versus multistep binding events.^11^ This level of resolution is essential for dissecting biological processes, including receptor–ligand recognition, antibody–antigen specificity, enzyme–substrate interactions, and allosteric regulation.^12^ Its applicability extends to complex systems such as lipid membranes, intact cells, and multiprotein assemblies, thereby providing insights into signal transduction, host–pathogen interactions, and regulatory networks. Compared to nuclear magnetic resonance, another technique used to study molecular interactions, SPR has several advantages, including label-free detection, the ability to use small amounts of various types of samples, high-throughput screening, and real-time monitoring of binding kinetics.^13^

Moreover, SPR biosensors are used to study conformational changes, since when a protein undergoes a structural change, the refractive index and optical thickness at the surface of the metal film are also modified. In this way, structural transitions can be monitored during protein-small molecule interactions.^14^

SPR has become an indispensable tool in modern drug discovery and development. This technique enables researchers to screen novel drug candidates, analyze their interactions with target proteins, and assess the pharmacokinetic and pharmacodynamic characteristics of lead compounds. By providing real-time information on binding kinetics, affinity, and specificity, SPR greatly accelerates the identification of potential therapeutics while reducing both time and resource requirements.^15^ In recent years, increasing attention in drug design has been directed toward the kinetic aspects of interactions, particularly the drug–target residence time, as it reflects the temporal dynamics of drug concentrations in vivo. SPR is now widely applied in biotherapeutic development, early drug discovery, and the study of protein stability and functionality during biopharmaceutical production.

The discovery of novel biotherapeutics, especially for oncology, remains a major challenge for the pharmaceutical industries. Consequently, there is a growing need for rapid analytical characterization tools capable of assessing biomolecules during early clinical development stages, a process facilitated by the use of biomarkers.^16^ In contrast to traditional methods such as ELISA, SPR does not require fluorescent or radioactive labeling, thereby simplifying sample preparation and eliminating potential alterations in molecular binding behavior.

SPR biosensor platforms are also increasingly utilized in High Throughput Screening (HTS) applications, particularly in multichannel formats where thousands of binding interactions can be continuously monitored on a single chip surface.^17^ As such, SPR has become a viable and often superior alternative to conventional HTS methods for identifying biologically active compounds that can serve as starting points for lead optimization.^15^ This biosensing technology enables the rapid detection of binding fragments and provides detailed kinetic data on biomolecular interactions. Parameters such as association and dissociation rates, affinity, and specificity can all be quantified, offering valuable insight into binding mechanisms and the structural factors influencing them. Unlike end point assays that yield only a single measurement, SPR generates dynamic, real-time data, revealing critical temporal information that would otherwise be lost.^13^

Beyond molecular analysis, SPR is increasingly employed to monitor cellular responses and physiological changes. The technique can detect cell-surface interactions and even identify the presence of tumor cells. When mammalian cells are exposed to reactive molecules, the resulting cellular responses manifest as variations in the SPR signal, corresponding to changes at the cell–molecule interface. Yanase et al.^18,19^ demonstrated that SPR sensors detect alterations in the refractive index within the evanescent field on the gold surface, meaning that the observed signals originate primarily from molecules located within or near the plasma membrane of cells attached to the chip. Consequently, enhanced cell adhesion, particularly in cell types that expand their contact area in response to external stimuli, produces a measurable shift in the refractive angle.

In tumor cell detection, monoclonal antibodies are often employed to recognize specific tumor-associated markers immobilized on the SPR sensor surface.^20^ Cells cultured directly on the chip respond to external stimuli, and their behavior is monitored in real time by the instrument. Wu et al.^21^ introduced an innovative approach for assessing live tumor cells treated with daunorubicin (DNR) using an SPR chip–cell interface. Their findings revealed that signal variations were closely linked to morphological and mass changes of the adherent cells as well as to refractive index shifts in the surrounding medium. The reduction in the SPR signal intensity correlated linearly with cell survival rates, suggesting that combining SPR with electrochemical analyses may offer a powerful method for evaluating the therapeutic efficacy of bioactive agents on living cells.

In modern clinical medicine, disease diagnosis and monitoring frequently rely on quantifying biomarkers in body fluids. These biomarkers comprise a diverse array of chemical and biological molecules whose altered concentrations or activities are indicative of specific pathological states. Clinical laboratories commonly employ ELISA, chemiluminescence immunoassays, or PCR to measure these biomarkers.^22^ While effective and versatile, such methods often require multiple steps, time-consuming procedures, extensive sample preparation, trained personnel, and costly equipment. Moreover, these conventional techniques are typically centralized in specialized laboratories, leading to delays between sample collection and diagnostic results, potentially affecting clinical outcomes and patient care.

Given its intrinsic advantages, SPR represents a highly promising alternative for biomedical and clinical analyses. The method is compatible with colored or opaque samples and does not necessitate molecular labeling with fluorescent or radioactive tags, thereby preserving the native activity and binding properties of the analytes. This feature allows the direct study of biological samples, such as cells and tissues, in conditions that closely resemble their natural environment, providing more accurate insights into in vivo molecular behavior.^23^ The past decade has witnessed a significant expansion in research focused on the development and application of SPR for biomolecular analysis. Numerous reports have demonstrated its successful use in detecting disease-related analytes in patient-derived clinical samples. Notably, while most detected molecules fall within nanomolar or nanogram per milliliter concentration ranges, several studies have achieved sensitivities at picomolar or even pg/mL levels. SPR has been effectively applied to a variety of biofluids including plasma, serum, whole blood, urine, and saliva. The continually expanding range of analytes measurable by SPR highlights its growing potential as a diagnostic and clinical tool.

#### 2.1.1 Tumor Markers

The detection and analysis of tumor-related and other disease biomarkers are crucial for predicting disease onset and reducing the need for invasive medical procedures that could negatively impact patient health. In this context, SPR biosensors have emerged as valuable analytical tools due to their high sensitivity, portability, low sample volume requirements, and ability to perform multiplexed detections.

SPR biosensors have demonstrated effectiveness in identifying tumor markers using various bioactive recognition elements, including antibodies, DNA and microRNA sequences, circulating proteins, exosomes, and lipids. Several representative studies illustrate their versatility and analytical performance.

Li et al.^24^ developed a real-time, highly sensitive SPR biosensor for detecting carcinoembryonic antigen (CEA) in human serum. The system employed two antibodies targeting distinct CEA epitopes with strong affinity and specificity. To enhance the detection signal, streptavidin-functionalized gold nanoparticles (AuNPs) were incorporated through biotin–streptavidin coupling. This AuNP-enhanced sandwich SPR biosensor successfully achieved a sensitive detection range of 1–60 ng/mL with a limit of detection (LOD) of 1000 pg/mL.

The SPR-based detection of cancer cells represents another promising avenue in clinical diagnostics, achievable through diverse sensor surface functionalization strategies. For instance, Chen et al.^25^ reported a magnetic nanoparticle-assisted SPR system for the detection of folic acid and MUC-1 biomarkers in breast cancer cells. Since the folate receptor is overexpressed in MCF-7 breast tumor cells, the researchers utilized MUC-1 functionalized with cysteine aptamer-linked, folic acid-bound magnetic nanoparticles to selectively capture these cells. The SPR angle shift correlated directly with the number of MCF-7 cells bound, confirming the specific cell capture on the MUC-1-modified surface. The biosensor achieved a detection limit of approximately 500 cells. Similarly, Zhu et al.^26^ introduced a three-dimensional multilayer SPR biosensor based on DNA hybridization for label-free detection of live lung tumor cells. This system featured asymmetric gold nanoholes and nanosquares integrated into a microfluidic platform, enabling the identification of lung tumor cells at concentrations as low as 10^–7^ M using only 2 μL of the sample.

Circulating proteins are also valuable biomarkers for cancer detection, as they can diffuse into the bloodstream from the tumor microenvironment. Using this principle, a sensor for detecting cytokeratin 19 (CK19), a lung cancer marker, was developed with graphene oxide modified by carboxyl groups attached to gold chips via cystamine.^27^ The sensor functionalized with anti-CK19 antibodies achieved an exceptional detection limit of 0.05 pg/mL.

Gold nanoparticles (AuNPs) are frequently used to improve biosensor sensitivity due to their unique optical properties. For example, an SPR-based hybrid colorimetric and plasmonic sensor was employed to detect prostate-specific antigen (PSA), a biomarker for prostate cancer, achieving a LOD of 9 pg/mL.^28^ In this approach, triangular AuNPs conjugated with PSA-binding antibodies were reacted with PSA molecules in the presence of magnetite nanoparticles coated with secondary antibodies. In another study, Kim et al.^29^ designed an optical fiber-based localized surface plasmon resonance (LSPR) sensor for rapid, direct quantification of thyroglobulin, a biomarker for monitoring thyroid cancer recurrence. Gold nanoparticles conjugated with antithyroglobulin antibodies were immobilized on an optical fiber coupled with a microfluidic channel, protecting the system from external exposure. The resulting biosensor demonstrated an impressive LOD of 0.09311 pg/mL, exhibiting excellent selectivity toward thyroglobulin.

Dysregulated microRNA (miRNA) expression is strongly associated with various cancers.^30^ Since miRNAs are stable in circulation, they serve as attractive biomarker targets. A novel enzyme-assisted target recycling method was proposed for detecting gastric cancer-associated miRNA (miR-10b) in plasma and urine, reaching an LOD of 2.45 pM.^31^ The detection process involved three main steps: (1) formation of a DNA sandwich structure via sequence-specific hybridization using Au nanotags coated with tannic acid-modified DNA; (2) enzymatic target recycling; and (3) enhancement of the LSPR signal. Similarly, miRNA-200 and miRNA-141 were identified in tumor cell extracts and serum using an SPR sensor integrating graphene oxide (GO)–AuNP multilayers. Mujica et al.^32^ fabricated a GO-enhanced SPR nanosensor for detecting miRNA-21, a prognostic biomarker for cervical cancer, achieving an extraordinarily low LOD of 0.0003 pM from urine samples. The device utilized a DNA probe covalently bound to poly­(diallyldimethylammonium chloride) (PDDA) and GO bilayers on a gold surface functionalized with 3-mercaptopropanesulfonate. The field-enhancing property of GO facilitated probe immobilization and improved the sensitivity. Furthermore, Zhang et al.^33^ reported a label-free LSPR nanoprobe using DNA-modified gold nanocubes (AuNCs) for detecting miRNA-205, a lung cancer biomarker. This technique allowed real-time monitoring of hybridization-induced dielectric changes, achieving an LOD of 5 pM in serum samples.

Exosomes, which carry molecular cargo reflecting the genetic and signaling profiles of their parent tumor cells, have also been investigated as potential targets for SPR biosensors.^34^ Because the size of the exosomes matches the sensing depth of SPR, label-free detection is often possible. For instance, a AuNP-based SPR aptasensor was developed to distinguish exosomes derived from MCF-10A (normal breast cells) and MCF-7 (breast cancer cells) in fetal bovine serum. Compared to conventional ELISA assays, this SPR-based approach achieved a 10^4^-fold lower LOD (5 × 10^3^ exosomes/mL) and successfully differentiated between the two cell types.^35^

Finally, alterations in lipid metabolism are hallmarks of many cancers, and lipid-based biomarkers are gaining prominence in diagnostics. Li et al.^36^ designed a double cross-linked supramolecular hydrogel (DCSH) as a host matrix for lysophosphatidic acid (LPA), a biomarker for early stage ovarian cancer. In this system, ferrocene and β-cyclodextrin (β-CD) served as the host–guest pair, while LPA acted as a competitive guest molecule that disrupted their interaction. Using SPR coupled with optical waveguide spectroscopy (SPR-OWS), the biosensor achieved selective and sensitive detection of LPA with an LOD of 122000 pM, demonstrating the potential of DCSH-based SPR-OWS sensors for LPA quantification in plasma-like samples.

#### 2.1.2 Hormones

Hormones are crucial signaling molecules that are produced by different glands in the body and carried through the bloodstream to specific organs, tissues, and cells. Imbalances in these processes can lead to disease. Because of their significant impact on human health, precise and rapid monitoring of hormones is essential.

Castiello et al.^37^ developed a multiplex surface plasmon resonance imaging (SPRi) biosensor capable of the simultaneous quantification of insulin, glucagon, and somatostatin. The sensor surface was functionalized with a mixed self-assembled monolayer (SAM) composed of a linear thiol terminated with a carboxyl group for hormone immobilization and a low-molecular-weight thiolated polyethylene glycol (CH_3_O–PEG–SH), which served as both a spacer and an antifouling agent. Covalent attachment of insulin, glucagon, and somatostatin was achieved through NHS/EDC coupling chemistry. For multiplex measurements, standard mixtures containing all three hormones (1–4000 ng/mL in PBS-T buffer) were combined with an antibody cocktail consisting of anti-insulin (1 μg/mL), antiglucagon (2 μg/mL), and antisomatostatin (2 μg/mL). The limits of detection (LOD) in multiplex mode were 1000 pM for insulin, 4000 pM for glucagon, and 246000 pM for somatostatin.

In another study, Cao et al.^38^ designed an indirect SPRi immunosensor for the quantitative detection of 17β-estradiol (E2), the most biologically active estrogenic hormone in humans and domestic animals and a key endocrine-disrupting compound. A BSA–E2 conjugate was immobilized on the SPR chip by coupling its primary amine group to a thiol-succinimide monolayer formed on the gold surface. During analysis, free E2 molecules in the sample competed with immobilized BSA–E2 for binding to a monoclonal anti-E2 antibody. The sensor achieved an impressive LOD of 0.3 pM, suitable for detecting physiological concentrations of E2.

A flexible localized surface plasmon resonance (LSPR) biosensor was introduced by Nan et al.^39^ for cortisol detection in human samples. The device was constructed by depositing gold nanoparticle layers onto a 3-aminopropyltriethoxysilane (APTES)-functionalized poly­(dimethylsiloxane) (PDMS) substrate. A cortisol-specific aptamer was immobilized on the gold nanoparticle surface, enabling selective hormone recognition. The sensor demonstrated excellent detection performance across a wide dynamic range of 100–1000000 pM, with a LOD of 100 pM, confirming its potential for physiological cortisol monitoring.

Faridli et al.^40^ developed an LSPR-based immunoassay for the detection of prolactin in human serum. Gold nanoparticles were synthesized and functionalized by electrostatic adsorption of antiprolactin antibodies onto their surfaces. The detection principle relied on monitoring LSPR peak shifts proportional to the prolactin antigen concentration. The biosensor achieved a linear dynamic range of 1000–40000 pg/mL, with an LOD of 800 pg/mL and a sensitivity of 10 pg/mL, demonstrating high precision in hormone quantification.

The Molecularly Imprinted Polymer (MIP) approach provides a robust method for creating synthetic recognition sites with high specificity. Using this strategy, Cenci et al.^41^ employed precipitation polymerization to synthesize a library of MIP nanoparticles (NPs) selective for the N-terminus of hepcidin-25, a hormone whose serum concentration is associated with iron metabolism disorders and doping detection. The biotinylated MIP NPs were immobilized on a NeutrAvidin-coated SPR sensor chip. The resulting sensor exhibited high affinity and selectivity for hepcidin-25, achieving an LOD of 5 pM, underscoring the capability of MIP-based SPR systems for highly specific hormone detection.

#### 2.1.3 Rare Disease Biomarkers

Rare diseases are characterized by a wide heterogeneity of symptoms and clinical manifestations, which not only differ between diseases but also vary significantly among individuals affected by the same condition. The limited medical expertise, scarcity of scientific knowledge, and insufficient healthcare resources dedicated to these disorders often result in misdiagnoses and delayed treatment. Moreover, since rare diseases frequently present with nonspecific or overlapping symptoms, their detection is particularly challenging. In this context, SPR biosensors offer a promising analytical platform for identifying disease-associated biomarkers due to their high sensitivity, label-free detection, and rapid analysis capabilities. Importantly, rare diseases not only burden patients but also exert substantial emotional, social, and economic impact on families, caregivers, and society as a whole.

A novel SPR-based immunoassay for the quantitative detection of D-dimer in human plasma was developed and validated by Hu et al.^42^ D-dimer, a fibrin degradation product generated during fibrinolysis, serves as an established biomarker for diagnosing thrombotic disorders. Using a Biacore T200 instrument, anti-D-dimer antibodies were covalently immobilized on the sensor chip through amine coupling. The system achieved a limit of detection (LOD) of 8300 pg/mL, enabling a sensitive and rapid analysis in clinical plasma samples.

Canovi et al.^43^ reported the development of an SPR immunoassay for human pentraxin-3 (PTX3), a key component of the pentraxin family involved in inflammatory and immune responses. PTX3 expression is induced in endothelial cells and macrophages during inflammation, while its structural analogue, C-reactive protein (CRP), is widely used as a biomarker of acute immune reactions. The assay employed a ProteOn XPR36 SPR platform, where anti-PTX3 antibodies were immobilized via amine coupling onto an alginate-modified gold surface. Both recombinant (rhPTX3) and endogenous plasma PTX3 were successfully detected, with a LOD of 5000 pg/mL, demonstrating the assay’s reproducibility and clinical applicability.

An advanced SPR biosensor utilizing a carboxyl-functionalized molybdenum disulfide (MoS_2_) film was designed by Chiu et al.^44^ to detect pregnancy-associated plasma protein-A2 (PAPP-A2), a biomarker linked to fetal Down syndrome. Anti-PAPP-A2 antibodies were covalently immobilized on the MoS_2_ surface through the amine groups of the lysine residues. Maternal serum samples from both normal and Down syndrome pregnancies were analyzed, and the biosensor exhibited remarkable sensitivity with a LOD of 0.05 pg/mL. The high affinity of MoS_2_ toward biomolecules and its strong biofunctionalization capacity highlight its potential for clinical diagnostic applications.

To enable the rapid and sensitive detection of thrombin, a crucial biomarker for coagulation and cardiovascular disorders, Cimen et al.^45^ fabricated a molecularly imprinted SPR biosensor. Thrombin-imprinted and nonimprinted nanoparticles were synthesized and immobilized on an allyl mercaptan-modified gold surface to create selective recognition sites. The sensor demonstrated an extremely low LOD of 0.017 pM in aqueous samples and 0.033 pM in patient serum, confirming its capacity for ultrasensitive and specific biomarker detection.

In a separate study, Vashist et al.^46^ developed a sensitive SPR immunoassay for human fetuin A (HFA), a glycoprotein implicated in atherosclerosis. The immobilization process involved diluting anti-HFA antibodies in 1% (v/v) 3-aminopropyltriethoxysilane (APTES) and applying them to a KOH-treated gold-coated chip. The assay enabled quantification of HFA concentrations in the range of 300–20000 pg/mL, with a LOD of 700 pg/mL and a sensitivity of 1000 pg/mL, illustrating both precision and efficiency in serum analysis.

Nangare et al.^47^ constructed a graphene oxide (GO)-chitosan (CS)-based SPR biosensor using a layer-by-layer (LbL) assembly technique for detecting beta-amyloid_1–42_ (Aβ_1–42_), a key biomarker for Alzheimer’s disease. Environmentally friendly synthesis of silver nanoparticles (AgNPs) and GO was employed. The multilayer structure (AgNPs–CS–PSS–CS) was functionalized with anti-Aβ antibodies, taking advantage of the CS amine groups for enhanced immobilization and orientation. The final GO-modified SPR chip achieved a detection range of 0.002–400000 pg/mL and an exceptionally low LOD of 0.00121 pg/mL, underscoring its potential in neurodegenerative disease diagnostics.

Recent technological advances have demonstrated the capability of SPR-based platforms to achieve ultrasensitive detection of biomarkers in cerebrospinal fluid (CSF). For instance, Zhang et al.^48^ developed an optical fiber SPR biosensor integrating a tilted fiber Bragg grating and a microfluidic multichannel system to differentiate monomeric and oligomeric forms of amyloid-β_42_ (Aβ_42_) in CSF, achieving detection limits in the tens of pg/mL range. Such sensitivity highlights SPR’s suitability for complex biological matrices and conformational biomarker analysis. The same sensing strategy could be extended to amyotrophic lateral sclerosis (ALS) diagnostics, targeting CSF biomarkers such as phosphorylated and light-chain neurofilaments (pNfH, NfL), superoxide dismutase 1 (SOD1), and TAR DNA-binding protein 43 (TDP-43), which exist at subpicogram concentrations.^49^

Our research group has also developed an SPRi-based analytical platform for the determination of erythropoietin (EPO) in biological fluids.^50^ EPO regulates erythroid precursor cell proliferation and serves as a therapeutic agent in various disorders. Moreover, elevated EPO activity has been observed in Alzheimer’s disease (AD). The SPRi chip, fabricated on a BK7 glass substrate coated with 1 nm titanium and 50 nm gold layers, was functionalized by immobilizing anti-EPO antibodies through amine coupling chemistry. The assay was optimized using calibration curves to establish the limits of detection (0.03 pg/mL) and quantification (0.10 pg/mL). Validation confirmed the method’s high precision, accuracy, and sensitivity, demonstrating its suitability for detecting trace EPO levels in clinical and research applications.

Table 1 summarizes the characteristics of the SPR biosensors mentioned above for biomedical applications.

**Table 1:** Overview of SPR Biosensors for Applications in Biomedicine

**Analyte**	**Type of probe**	**Clinical sample**	**LOD**	**Reference**
Carcinoembryonic Antigen (CEA)	Fluidic SPR	Serum	1000 pg/mL	^24^
MCF-7 cells	SPR	Serum	500 cells/mL	^25^
Lung tumor cells	SPR	Serum	10^–7^ M	^26^
MiRNA-10b	LSPR	Plasma, urine	2.45 pM	^31^
MiRNA-21	SPR	Urine	0.0003 pM	^32^
MiRNA-205	LSPR	Serum	5 pM	^33^
Cytokeratin 19	Fluidic SPR	Serum	0.05 pg/mL	^27^
PSA	SPR	Serum	9 pg/mL	^28^
Thyroglobulin (Tg)	LSPR	Serum	0.09311 fg/mL	^29^
Exosomes generated by MCF-10A and MCF-7	SPR	Fetal bovine serum	5 × 10^3^ exosomes/mL	^30^
Lysophosphatidic Acid (LPA)	SPR	Blood plasma	122000 pM	^36^
Insulin, glucagon, and somatostatin	SPRi	Aqueous solutions	1000 pM, 4000 pM, 246000 pM	^37^
17β-estradiol (E2)	SPRi	-	0.3 pM	^38^
Cortisol	LSPR	Sweat	100 pM	^39^
Hepcidin-25	SPR	Serum	5 pM	^41^
Prolactin	LSPR	Human serum	800 pg/mL	^40^
D-Dimer	SPR	Human plasma	8300 pg/mL	^42^
Pentraxin-3 (PTX3)	SPR	Human plasma	5000 pg/mL	^43^
Pregnancy-associated plasma protein-A2 PAPP-A2	SPR	Maternal serum	0.05 pg/mL	^44^
Thrombin	SPR	Aqueous solution, serum samples	0.017 pM–0.033 pM	^45^
Human fetuin A (HFA)	SPR	Human blood and plasma	700 pg/mL	^46^
Beta-amyloid_1–42_ (Aβ_1–42_)	SPR	Animal blood	0.00121 pg/mL	^47^
EPO	SPRi	Plasma	0.03 pg/mL	^50^

### 2.2 Limitations

The biosensors listed above are distinguished by very low LOD values and efficient receptor and analyte coupling methodologies, which help to increase the sensitivity. Nevertheless, they have limitations that still hinder their use in clinical settings. Translating SPR spectroscopy from research laboratories to clinical practice for disease biomarker detection remains challenging, primarily due to the difficulty in reproducing the low limits of detection (LOD) achieved under idealized conditions when analyzing real biological samples. The major limitations arise from matrix effects and biofouling, caused by the nonspecific adsorption of abundant serum proteins and lipids onto the sensing surface, which increases background noise and reduces the signal-to-noise ratio.^51^ Additional complications include preanalytical variability in sample handling, differences in refractive index and viscosity between buffers and biological fluids, and the intrinsically low abundance of biomarkers, often present at pg/mL levels.^52^ Other critical factors comprise mass-transport limitations, random orientation or denaturation of immobilized ligands, difficulties in surface regeneration without loss of activity, and instrumental drift induced by temperature and environmental fluctuations.^53^ The absence of matrix-matched reference standards and uniform calibration protocols further hampers interlaboratory reproducibility, while the structural heterogeneity of biomarkers, such as isoforms, complexes, or degradation products, can compromise analytical specificity.

Although gold is commonly used as the preferred metal for coating sensor surfaces, in the case of repeated measurements, it is easily prone to be damaged. The use of other materials to reinforce the coating would improve the adhesion of the gold layer and increase the durability. One example is the use of polymerized allylamine.^54^ The robustness of the instrument itself can also be improved by eliminating spectral analysis and moving parts, using a tunable laser working at standard excitation and readout incidence.^55^

One of the main limitations of SPR-based biosensors lies in nonspecific binding when analyzing complex biological matrices, such as blood. In addition, cross-reactivity among multiple biomarkers within the same sample can lead to signal interference, resulting in false positive or negative outcomes that compromise detection accuracy and hinder clinical application. To address these challenges, antifouling surface modifications are employed. These coatings form a protective barrier that minimizes nonspecific adsorption of proteins through mechanisms like steric hindrance or hydration layer formation, thereby improving assay reliability. Commonly utilized antifouling agents include polyethylene glycol (PEG),^37^ zwitterionic polymers, and polysaccharides, which are typically grafted onto the gold layer of the SPR sensor to enhance its selectivity, sensitivity, and overall analytical performance.

Considering the examples of biosensors mentioned above, one limitation that seems to have been largely overcome concerns the reproducibility of SPR analyses. It is apparent that amide coupling is the most widely used procedure for binding ligands to the surface of sensors and that protocols are standardized, allowing for the accurate reproducibility of measurements. To carry this reproducibility of data over into clinical trials, implementation of robust analytical instrument qualification and quality assurance protocols is needed as well as performing thorough calibration procedures using reference samples and internal standards.

When it comes to sensor regeneration, the main problems encountered relate to the degradation or denaturation of ligands on the sensor,^56^ perhaps due to overly aggressive regeneration buffers, irreversibly binding analytes, or contaminants. The most effective solutions involve the use of buffers with optimized pH and salt concentrations^57^ or reversible coupling chemistries.

One of the most significant challenges is regulatory validation, which demands rigorous demonstration of analytical and clinical performance according to international standards such as ISO 13485, CLSI EP guidelines, and regulatory frameworks established by agencies like the FDA and EMA. Achieving regulatory approval requires not only proof of sensitivity, specificity, and reproducibility under controlled conditions but also verification of robustness and stability under real-world clinical workflows, which often involve variable environmental conditions, diverse operators, and heterogeneous sample matrices.^58^ Moreover, SPR devices are traditionally optimized for expert use in research laboratories with complex optical alignment, surface functionalization, and fluidic control systems that are incompatible with the simplicity, automation, and robustness expected in clinical POC instruments.

Another major barrier concerns the user interface and operational simplicity. Clinicians and laboratory technicians require devices that integrate automated calibration, error detection, and real-time data interpretation without the need for specialized training in optical biosensing. Current SPR systems often lack standardized cartridges, plug-and-play sensor chips, or integrated fluidics capable of handling unprocessed biological samples such as serum, plasma, or whole blood.^58^ Furthermore, interinstrument variability and the absence of harmonized performance criteria make multicenter validation studies difficult, delaying clinical translation. Bridging this gap requires multidisciplinary collaboration among engineers, clinicians, and regulatory experts to develop user-friendly, disposable SPR platforms with self-contained reagents, microfluidic automation, and standardized analytical protocols.

## 3 SPR Affinity Biosensors

In essence, SPR sensors function as thin-film refractometers capable of detecting variations in the refractive index that occur at the interface between a metal film and an adjacent dielectric medium. These changes are monitored through the evanescent field of the SP, which extends into the medium in contact with the metallic surface. Any alteration in the refractive index at the metal–dielectric interface, typically caused by biorecognition events, modifies the propagation constant of the SP, leading to a perturbation in the coupling conditions (such as the resonance angle, wavelength, intensity, or phase).^59^

An SPR affinity biosensor is composed of two key elements: a biorecognition layer and an optical transducer. The biorecognition layer, immobilized on the metal surface (often termed the sensor chip), contains specific ligands that interact with target analytes in solution. When analyte molecules bind to these immobilized receptors, the local refractive index at the sensor surface increases, and this variation is detected optically by the transducer as a measurable SPR signal.

The magnitude of the refractive index change, and thus the intensity of the SPR response, depends on both the surface concentration of the bound analyte and the intrinsic optical properties of the molecules. When binding occurs within a thin surface layer of thickness h, the SPR response is directly proportional to the refractive index increment of the analyte, denoted as (d*n*/d*c*) (typically 0.1–0.3 mL/g for protein species), and the surface mass density, Γ, according to the established relationship.^59^

1

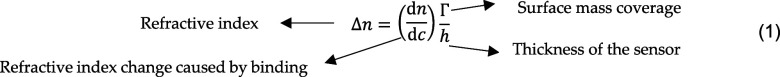




Thus, the refractive index increase is proportional to the surface mass coverage Γ. For this reason, the usual outputs of the current commercial instrumentation are the kinetics of the binding reactions, that directly express the mass coverage, in units 1 Resonance Unit (RU) = 1 pg/mm^2^, measured during the binding reaction.

### 3.1 Chemical Immobilization of Biomolecules

Immobilization of biomolecules on SPR sensor surfaces can be achieved through either covalent or noncovalent interactions, with covalent coupling being one of the most widely employed approaches.^1^ In this method, the sensor chip surface is first activated with chemical reagents to generate reactive functional groups, which then form stable covalent bonds with complementary groups on the biomolecule.

Chemical cross-linkers with spacer arms are commonly used to facilitate this process. Typically, the immobilized biomolecule contains two functional groups: one for covalent attachment to the sensor surface and another to preserve the molecular functionality. A variety of protein–linker conjugates have been developed to optimize immobilization efficiency.^60^ Functional groups such as thiols, amines, and aldehydes are frequently employed for covalent attachment (Figure 2). For instance, thiol groups in cysteine residues can react with heterobifunctional cross-linkers like maleimide, which is a standard strategy for protein immobilization.^61^

**Figure 2:**
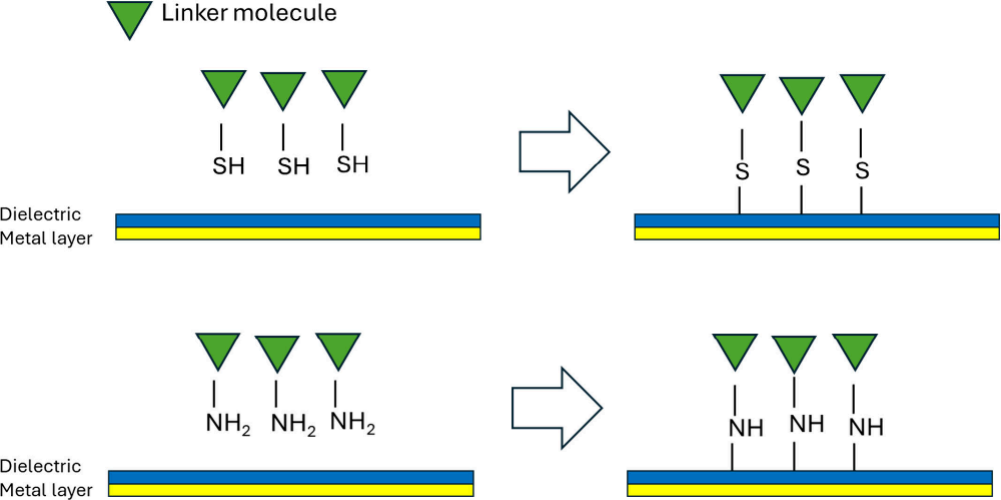
Schematic illustration of immobilization of chemical cross-linkers through thiol and amine functional groups.

Covalent coupling offers several advantages, including stable attachment, simplicity, and the ability to functionalize the surface without modifying the ligand. However, there are notable limitations. Covalent immobilization can potentially alter the active sites of proteins, affecting their binding activity. Moreover, reactive groups on the surface can be blocked by nonspecific protein adsorption, and the use of inappropriate blocking agents may inactivate the immobilized biomolecules.

The immobilization of biomolecules can also occur through an affinity capturing strategy, i.e., the modification of the surface of the chip in order to capture special proteins conjugated with a tag. Ligands are installed on the surface and function as bait, attracting their targets, such as proteins fused with an affinity tag. An example of these interactions is the affinity that exists between biotin and streptavidin, a widely used method of noncovalent affinity immobilization of biomolecules. Streptavidin-labeled proteins can interact with a biotin-functionalized surface or can act as a bridge between the biotin layer and biotinylated biomolecules (Figure 3).^62^ Unidirectional immobilization is employed to prevent the formation of randomly oriented biomolecular complexes on the sensor surface, which can occur during conventional covalent coupling. By ensuring a controlled and uniform orientation, this strategy enhances the sensitivity and specificity of the resulting SPR biosensor. A widely used approach relies on the strong affinity between gold surfaces and sulfur-containing biomolecules, a principle central to unidirectional immobilization. The formation of self-assembled monolayers (SAMs) through thiol (−SH) groups enables an orderly molecular orientation and consistent binding. In practice, cysteine residues in proteins or peptides are exploited to anchor biomolecules onto the gold surface of the SPR sensor.^63^ This direct immobilization method offers several advantages, including rapid preparation, simplicity, favorable molecular orientation, and enhanced functional activity of the immobilized biomolecules.

**Figure 3:**
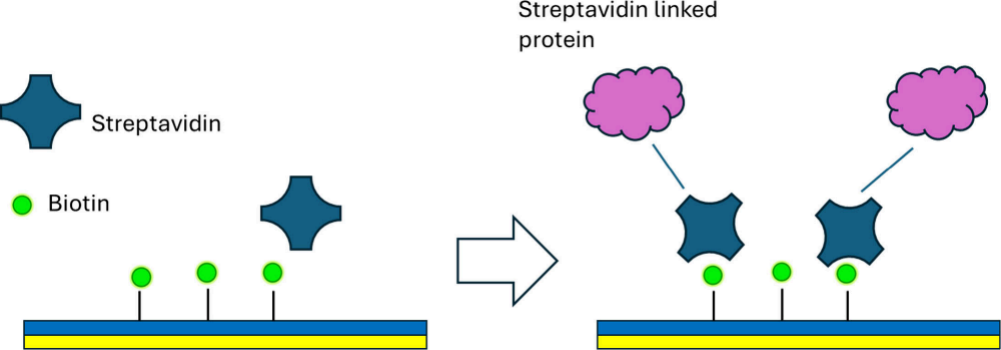
Example of the affinity capturing method through the interaction of biotin and streptavidin.

The thiol-gold bond has always been considered by the scientific community to be a very strong covalent bond that can be conveniently exploited in the context of SPR biosensors to anchor ligands to the surface of the chip. In recent years, however, studies have emerged that suggest that the thiol-gold bond is not as strong as previously thought.

Inkpen et al.^64^ performed an experiment on thiolated SAMs to measure the conductance of the single-molecule junction of the thiol-gold bond and observe the fate of the thiol hydrogen. The measurements, carried out using the STM break junction technique, showed that the hydrogen atom is not lost from the thiol when it binds to the gold. This is because if the hydrogen is not lost, it means that in the thiol-gold interaction there is no significant change in the chemical structure of the molecule interacting with the gold surface. Thus, Inkpen and co-workers assume that these thiolated SAMs do not predominantly form covalent bonds with gold. This implies that thiolated molecules on the gold surface possess a high mobility, given the lability of the thiol-gold bond, and that van der Waals interactions play a non-negligible role in providing stability to the adsorbed layers.

The immobilization of biorecognition elements on the SPR sensor surface is a critical factor that directly influences both the analytical performance and the operational lifetime of the device. Conventional covalent coupling using EDC/NHS activation of carboxyl-terminated SAMs remains the industry standard, owing to its robustness, reproducibility, and broad compatibility with diverse ligands.^65^ Nevertheless, this approach can result in random ligand orientation and the potential inactivation of functional sites, which may compromise sensor performance. To address these limitations, site-specific covalent strategies have been developed, including thiol–maleimide coupling, vinyl sulfone addition, and copper-free click chemistry. These methods preserve the biological activity of immobilized molecules while enhancing surface stability and overall sensor reliability.^66^

Recently, covalent peptide-based systems such as SpyTag/SpyCatcher and Sortase A-mediated conjugation have emerged as particularly promising approaches, enabling precise, orientation-controlled immobilization with remarkable resistance to regeneration cycles.^67^ These bioorthogonal chemistries form spontaneous or enzymatically catalyzed isopeptide bonds, resulting in near-permanent anchoring of proteins under physiological or mildly denaturing conditions. In parallel, surfaces functionalized with Protein A/G covalently bound to PEGylated SAMs offer a semiregenerable platform: antibodies can be noncovalently captured and replaced without compromising the stability of the underlying interface.^68^

Recent advances in surface chemistry have significantly weakened the performance of SPR biosensors in complex biological matrices. Zwitterionic polymers, including sulfobetaine, carboxybetaine, and poly­(carboxybetaine methacrylate) (PCBMA), have emerged as highly effective antifouling coatings, drastically reducing nonspecific protein adsorption and baseline drift in serum or plasma.^69^ Hydrophilic zwitterionic hydrogels and “self-defensive” coatings further facilitate analyte diffusion while minimizing surface fouling, enabling repeated measurement cycles with retained sensor activity.^70^ In parallel, N-heterocyclic carbene (NHC) SAMs on gold have demonstrated superior chemical stability compared to conventional thiol-based SAMs, leading to enhanced shelf life and reduced desorption under variable pH and ionic strength.^71^ Polymer brushes grown in situ create dense antifouling layers; while thick brushes can slightly attenuate the evanescent SPR field, careful optimization preserves sensitivity for analytes near the surface.^72^ Collectively, these strategies have been shown to reduce nonspecific adsorption by over 90% in challenging biological fluids and allow tens to hundreds of measurement cycles with minimal baseline drift, representing a critical step toward clinically deployable, reproducible, and reusable SPR biosensors.

#### 3.1.1 Typical Structure of Biomolecules Immobilized on the Gold Chip

A wide range of macromolecules can be immobilized onto the gold surface of an SPR chip, including antibodies, nucleic acids, synthetic receptors such as molecularly imprinted polymers (MIPs), aptamers, artificial DNA constructs, and various peptides and proteins.^73^

Nucleic acids, which are long-chain polynucleotides composed of repeating nucleotide units, each consisting of a nitrogenous base, a pentose sugar, and a phosphate group, are commonly immobilized after chemical modification with thiol groups or through biotinylation, enabling affinity interactions with avidin or streptavidin coatings on the chip surface (Figure 4a).^74^ In both strategies, the formation of a SAM enhances the flexibility of the bioreceptor, promoting more effective analyte binding. Additionally, nucleic acids can be linked to proteins or antibodies for site-specific immobilization through sequence-directed hybridization between a thiolated single-stranded DNA and its complementary strand anchored to the gold surface, a method particularly suitable for multichannel sensor arrays.^75^

**Figure 4:**
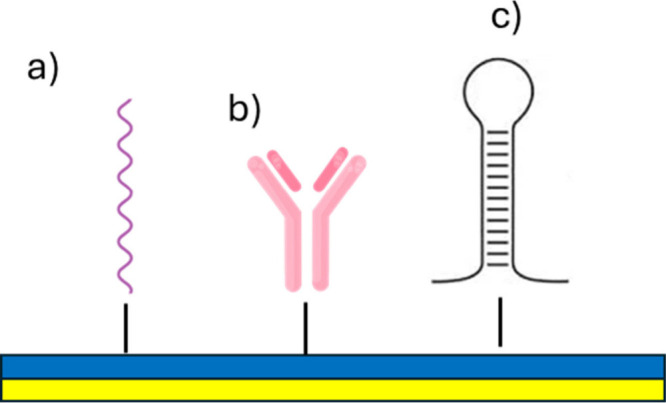
Illustration of types of biomolecules that can be immobilized on the gold chip. a) Nucleic acid (RNA, DNA). b) Antibody. c) Aptamer.

Antibodies, composed of two heavy chains and two light chains forming a Y-shaped structure with distinct Fab and Fc regions as well as proteins and peptides, are typically immobilized by covalent amide coupling or via biotin–streptavidin interactions to preserve conformational flexibility (Figure 4b). For antibodies, oriented immobilization can be achieved by exploiting the specific interaction between the Fc region and protein A or protein G, as demonstrated by Hirlekar Schmid et al.^76^ In this configuration, protein A is covalently attached to the surface using a homobifunctional cross-linker, forming a protective layer that prevents nonspecific adsorption while ensuring that the Fc region binds selectively, thus maintaining the antibody’s active binding sites in an accessible orientation.^77^

Aptamers, which are short nucleic acid sequences capable of binding to a wide range of targets—including small molecules, peptides, and proteins, with high affinity and specificity, represent another valuable class of recognition elements (Figure 4c).^78^ They offer several advantages over antibodies: thermal and chemical stability, low production cost, and reusability without significant loss of performance.^79,80^ Due to their small size, aptamers can achieve high surface density and multivalent binding. They can be immobilized on the gold surface through thiol-alkane linkers forming a SAM, or via biotin–avidin coupling, as shown by Wu et al.^81^ for the detection of aflatoxin in vinegar. In another approach, Wang et al.^82^ designed a three-dimensional tetrahedral DNA nanostructure bearing an aptamer probe at the apex and three biotinylated anchoring sites at the base. This nanostructure, immobilized on the gold chip through biotin–streptavidin binding, provided a highly ordered orientation, controlled spacing, and enhanced structural stability of the aptamer layer, leading to improved sensor performance.

## 4 Detection Formats

In SPR biosensors, several detection formats have been developed to ensure that the binding interactions occurring at the sensor surface generate a measurable and quantifiable signal.^83^ The three most widely used configurations are the direct detection, sandwich, and competitive inhibition assays.^84–86^

The direct detection format is typically employed when the analyte concentration is sufficient to produce a detectable change in the refractive index (Figure 5). In this approach, the analyte present in the sample binds directly to a biorecognition element immobilized on the sensor surface, and the resulting variation in the refractive index is proportional to the analyte concentration. While this method is straightforward and label-free, its sensitivity can be limited when dealing with low-abundance analytes. In such cases, sandwich or competitive inhibition assays are often preferred as they enable signal amplification and enhanced detection limits, thereby expanding the analytical capabilities of SPR-based biosensing systems.

**Figure 5:**
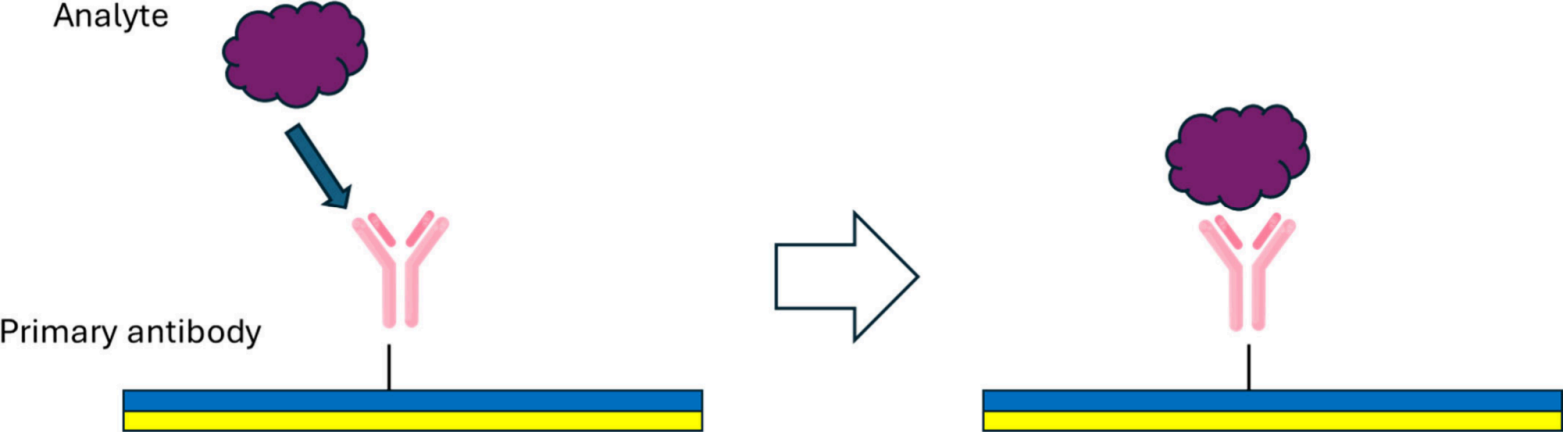
Schematic figure of the direct detection assay. The primary antibody is bound to the sensor surface and binds to the analyte.

In the sandwich assay format, detection occurs through a two-step binding process (Figure 6). Initially, the sample containing the analyte is introduced to the sensor surface, where the analyte molecules specifically bind to the capture antibodies immobilized on the sensor. In the second step, the sensor surface is exposed to a solution containing secondary antibodies, which recognize and bind to the analyte already attached to the primary antibodies. This dual binding event increases the total mass on the sensor surface, thereby amplifying the refractive index change and enhancing the sensor response. The resulting signal is directly proportional to the analyte concentration. However, this format is generally unsuitable for low-molecular-weight analytes, as their small size prevents simultaneous binding to both the capture and detection antibodies, making it difficult to form a stable sandwich complex.

**Figure 6:**
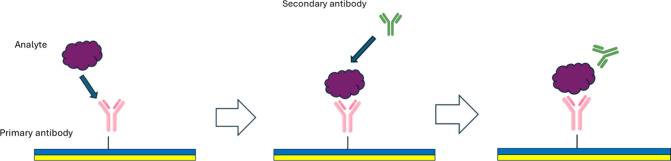
Sandwich assay. The primary antibody binds to the analyte, and the secondary antibody binds to the previously captured analyte.

The competitive inhibition assay format is particularly advantageous for the detection of low-molecular-weight analytes (Figure 7). In this approach, the analyte molecules are first preincubated with their corresponding antibodies, and the resulting mixture is then introduced onto a sensor surface coated with immobilized analyte molecules. The antibody concentration is maintained constant so that the observed response varies inversely with the analyte concentration in the sample. When the equilibrium mixture flows over the sensor, only unbound antibodies can interact with the immobilized analytes, leading to a decrease in signal intensity as the analyte concentration increases.

**Figure 7:**
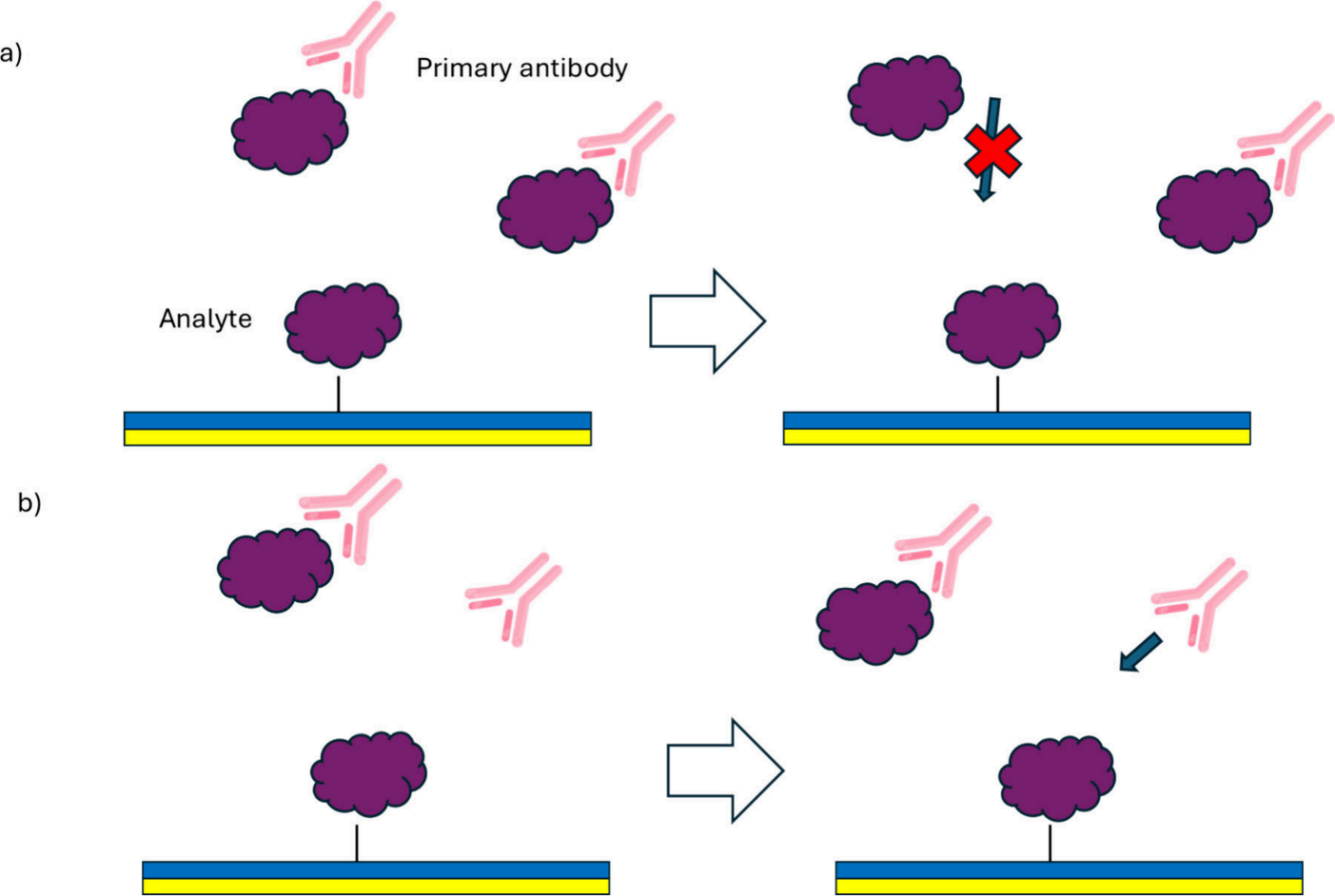
Competitive inhibition assay. a) The analytes are first mixed with a fixed concentration of antibodies, and the resulting mixture is then introduced onto a sensor surface coated with immobilized analyte molecules. At high analyte concentrations, most antibody binding sites are occupied, reducing the antibody availability for interaction with the surface-bound analytes. b) Conversely, at low analyte concentrations, more unbound antibodies are available to bind with the immobilized analytes on the sensor surface, leading to an increased sensor response.

Since the resulting change in the SPR signal is typically small, signal amplification strategies are often employed. This can be achieved by introducing a secondary binding molecule carrying a high molecular weight or high refractive index label, which enhances the optical response. Various amplification tags have been explored including liposomes, latex particles, and specific proteins. More recently, metallic nanoparticles (NPs) such as gold (AuNPs),^87^ palladium (PdNPs),^88^ and platinum (PtNPs)^89^ have been effectively utilized to improve the sensitivity of SPR biosensors. These nanoparticles have been applied to a wide range of assays, including antigen–antibody interactions, DNA hybridization,^90^ aptamer–target binding,^91^ and enzymatic reactions,^92^ demonstrating their versatility in enhancing SPR-based detection systems.

### 4.1 Metals for SPR Biosensors

Noble metals are generally used for the excitation of SPs in the Vis-NIR spectral region.

Indeed, the existence condition of SPs requires that the real part of the dielectric constant must be negative, while the narrower resonance width, linked to the resolution of the SPR detection, requires that |Re *e*_m_| ≫ Im *e*_m_. Both of these conditions are well satisfied mainly by silver and gold at frequencies below the plasma frequency.

Silver is the most potentially efficient metal for coating the surface of SPR sensors, as it has narrow resonance dips and therefore improves sensor resolution, but is chemically unstable and oxidizes easily.^93^ Copper as a metal coating has good conductivity but it also oxidizes easily, as does aluminum.^94^ Gold is the most common metal used to cover the surface of SPR biosensors due to its characteristics of biocompatibility, chemical stability, and variation in resonance, even if it has broader resonance peaks. It is widely used for its property of immobilizing a vast range of biomolecules that behave as ligands, without affecting their bioactivity.^95^ In recent years, biosensors based on graphene in its various forms have become widespread, thanks to its particular properties, which include the high electron transfer speed, the high mobility of the charge carriers, the low level of electrical noise, and the high density of active sites for the anchoring of chemical and biological species. The high optical transparency of the graphene monolayer makes it excellent for increasing the performance of SPR biosensors. It is an excellent surface for immobilizing biomolecules thanks to the presence of hydrophobic domains and π-systems. On the other hand, it still presents uncertainties regarding nonspecific interactions.^96^

### 4.2 Fluidic and Nonfluidic Biosensors

Depending on the measurement environment, SPR can be performed in two main configurations: fluidic and nonfluidic.^97^ In the traditional fluidic SPR setup, the biosensor assembly and measurement occur simultaneously in the presence of liquid media. This configuration is the most widely adopted in analytical applications, as the sample continuously flows through a measurement cell, while the optical signal is monitored in real time. During the assay, the biosensor surface is regenerated between measurements using a suitable cleaning solution, allowing multiple analyses on the same chip.^98^ Fluidic SPR enables precise monitoring of analyte–ligand interactions and provides real-time kinetic data on association and dissociation processes. However, this approach is relatively time-consuming and typically requires larger sample volumes, which can limit its suitability for certain applications.

In the nonfluidic system, the biosensor is formed ex situ and measurements are performed after removal of excess fluids. The nonfluidic method is typically performed in a stationary setup with a number of separate measurement points to increase the accuracy of the result. Multiple measurements can also be performed, and the chips can be regenerated. In the case of nonfluidic SPR, the biosensor is gently dried.^99^ Nonfluidic SPR allows the use of minimal amounts of sample, but as association kinetics cannot be observed, the efficiency of binding between ligand and analyte may be reduced.

### 4.3 Performances Indicators of SPR Sensors

Regardless of the excitation method employed, the underlying principle of SPR is that light propagating along a metal–dielectric interface enables biomolecular detection through monitoring shifts in the resonance spectrum.^100^ This phenomenon occurs when additional momentum is provided to the incident light to excite the surface plasmons. Consequently, the design and optimization of the SPR device are crucial to achieving an optimal sensor performance. Device optimization typically considers several key parameters, including sensitivity, resolution, LOD, figure of merit (FOM), and response time (RT). Sensitivity (S) is defined as the change in the optical signal with respect to variations in the refractive index of the substrate and can be divided into two components: 1) surface refractive index sensitivity, which depends on the interrogation method and reflects changes in the refractive index at the functionalized sensor surface due to specific biochemical interactions between receptor and analyte molecules; 2) bulk refractive index sensitivity, which depends on variations in the effective refractive index arising from changes in the refractive index of the medium surrounding the sensing region (as described in eq 8 from ref 59). The former represents localized changes at the sensor interface caused by analyte binding, whereas the latter describes refractive index fluctuations in the surrounding medium and defines the intrinsic sensitivity of the optical refractometer. Resolution is the smallest detectable variation in the surface refractive index that a sensor can resolve. It is typically expressed as ± 3σ, where σ represents the overall root-mean-square (RMS) noise of the measurement, quantified in either mass units or refractive index units (RIU). The refractive index resolution can therefore be expressed as
2

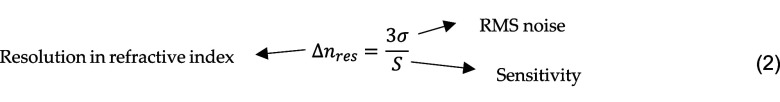




The LOD is the minimum analyte concentration that can be detected and can be expressed in terms of the concentration value (*C*) of the analyte under investigation, such as g/L or molarity (*M*).

Indicating as *S*_c_ the sensitivity of the output signal to the analyte concentration, and with σ_c_ the rms noise of the measurement, one can express the LOD via a relationship similar to the eq 2:^101^

3

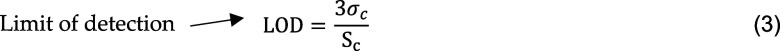




Alternatively, the LOD can be also calculated by using the resolution in the mass surface coverage, that is connected with the bulk refractive index resolution via eq 13 in ref 59. Another convenient measure of the overall sensor performance is the FOM, defined as S/fwhm, where S is the sensitivity and fwhm is the Full Wavelength at Half Maximum of the sensor output. This relationship can be applied to all the interrogation formats, and is strictly related to the signal-to-noise ratio (S/N) of the sensor device (eq 11 from ref 59). RT is a parameter representing the time the instrument takes to give a signal of distinguishable intensity from the background noise. Today’s commercially available SPR devices have some characteristic RTs. For example, the instrument by Gifford Bioscence Limited guarantees a limit of resolution of 0.5 RU in 10 s, while in approximately the same time Biacore S200 exhibits a resolution of 0.1 RU.^102^ BiOptix 404pi SPR has a resolution of ≈2 RU after 15 s acquisition, while Nicoya Lifetechnologies’ Open SPR takes ≈35 s^103^ to reach approximately the same coverage resolution. All of these instrumental parameters contribute to increasing the clinical relevance of measurements made with SPR instruments. High sensitivity and resolution coincide with the possibility of detecting trace elements, for example, in the early stages of a specific disease, when the levels of the biomarkers being sought are still low and cannot be detected using common diagnostic techniques. Wang et al.^104^ have developed an device with a very high FOM range (more than 1000 RIU^–1^), which could be of interest for future clinical applications.

With advancements in fabrication technologies, a wide variety of innovative prism, grating, and optical fiber configurations have been developed and integrated into SPR biosensors. By carefully designing the optical path and geometry of the prism coupling system, it is possible to achieve miniaturized sensor setups with a broad dynamic detection range. Similarly, optimizing parameters such as the substrate thickness in sinusoidal or rectangular grating structures as well as incorporating novel grating designs can lead to significant improvements in sensitivity and measurement range. In the case of fiber-based SPR sensors, enhanced sensitivity and strong resistance to noise can be obtained by tailoring the fiber substrate architecture. Examples include high-order cladding pattern designs,^105^ fusion-spliced fibers with intentional core mismatches,^106^ and microstructured fibers featuring air-hole arrays or asymmetric configurations.^107^ These structural innovations collectively contribute to improved detection performance and robustness in complex sensing environments.

### 4.4 Coupling Structures of SPR Sensors

In recent years, the advancement of prism coupling and precision processing techniques has driven the evolution of SPR prism devices toward miniaturization and portability. Traditional prism-coupled sensors rely on long optical paths, so achieving compact designs requires re-engineering components such as turntables and goniometers. To address this, Devanarayanan et al.^108^ introduced an innovative optomechanical scanning mechanism that replaces conventional goniometers with rotating mirrors and quadrant photodiodes, thereby simplifying the system design and improving manufacturability.

For prism-coupled SPR systems, two main challenges remain: simplifying the optical setup and expanding the detection range. The system can be streamlined by modifying the turntable geometry or substituting it with focusing lenses or rotating reflectors. Likewise, improving the incident optical path to increase the angle range can effectively broaden the sensor’s detection range. However, despite these innovations, prism-based systems remain bulkier and more expensive than fiber- and submicrometer grating-based SPR configurations. Moreover, the current simplified prism systems still face difficulties in combining miniaturization with a large dynamic detection range. Thus, realizing a fully miniaturized prism-coupled SPR platform requires the development of new evanescent wave coupling mechanisms capable of maintaining a wide detection range while reducing device size.^109^

Unlike prism-based designs, waveguide-coupled SPR sensors have seen limited progress in recent years. Their structural variability and lack of standardization have hindered consistent development, making miniaturization efforts in prism-coupled sensors the more active research direction. Indeed, only a few studies in the past decade have reported significant advances in waveguide-based SPR systems.

Meanwhile, grating-coupled SPR sensors have attracted considerable attention due to their tunable structural flexibility, which allows both sensitivity and the detection range to be adjusted by design. Because the grating performance strongly depends on its geometric configuration, optimizing the grating structure is crucial for enhancing the signal response. Recent literature indicates that simple grating geometries are amenable to miniaturization and mass production; however, nonfiber grating structures are often limited by fabrication constraints. Consequently, many proposed designs remain at the simulation stage, and the balance among structural complexity, manufacturability, and sensor performance continues to be a central challenge.

In summary, SPR substrate architectures can be broadly categorized into three coupling configurations (Figure 8): prism-coupled structures, which can achieve an extended detection range through optical path optimization and focusing lenses but remain costly and difficult to miniaturize without compromising precision; grating-coupled structures, where careful design of sinusoidal or rectangular gratings enhances sensitivity and accuracy, though fabrication remains complex and expensive; waveguide-coupled structures, which offer compact, integrable formats suitable for lab-on-chip applications, although their development is still in an early and fragmented stage.

**Figure 8:**
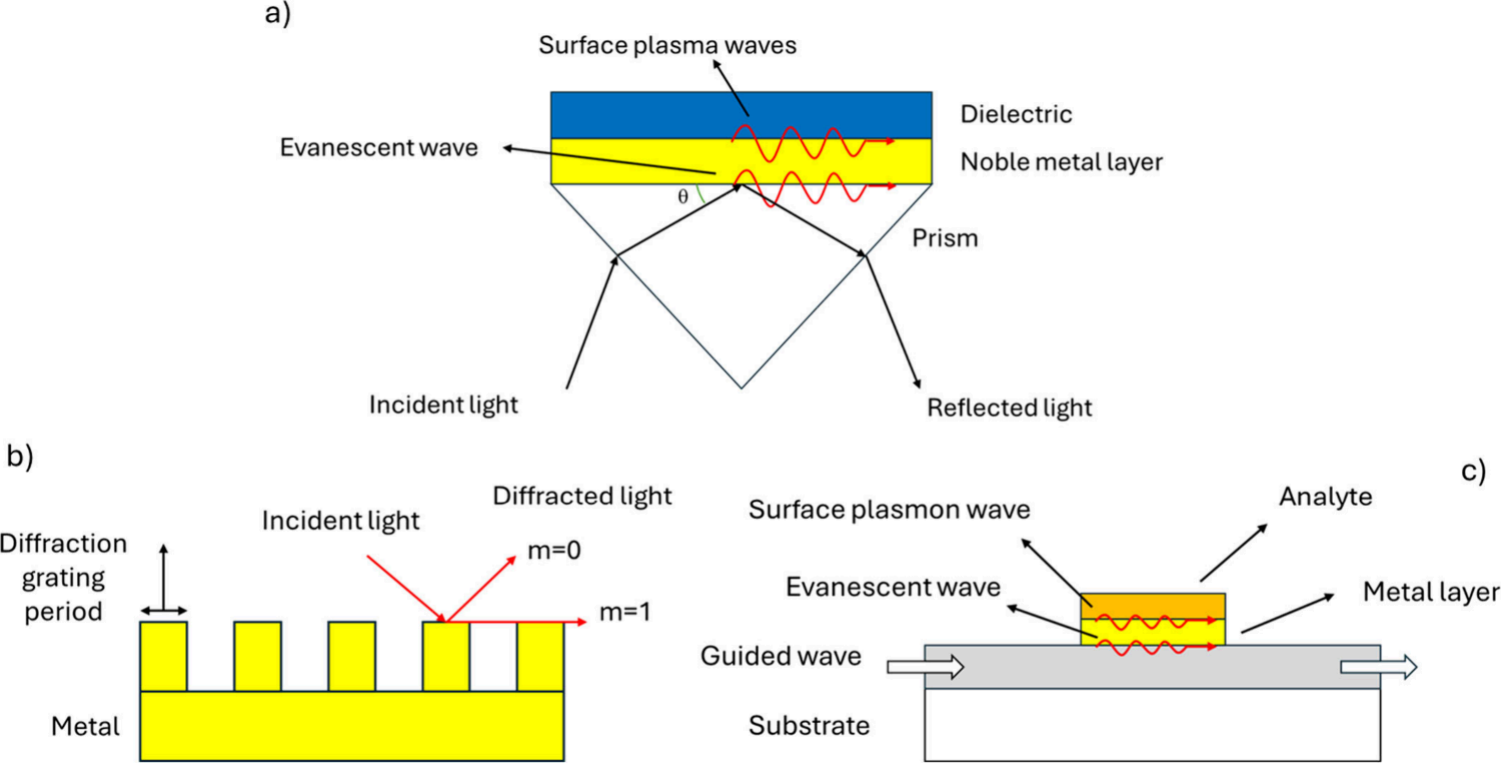
Scheme of coupling structures for SPR sensors: a) Prism-coupled structure: An evanescent wave is generated at the interface between the prism and the metal layer, penetrating the metal and exciting SPs at the interface between the metal and analyte layer. When the incidence angle and wavelength satisfy the resonance condition, SPs absorb the incident light efficiently, resulting in a dip in the reflected light intensity at a specific angle or wavelength in the angular reflection spectrum. b) Grating-coupled structure: SPs can also be excited by using a metallic diffraction grating, which provides the extra momentum required to match the SP wavevector. The incident light is diffracted into multiple orders, and coupling to the SP mode leads to a reduction in the specularly reflected light intensity at resonance. c) Waveguide-coupled structure: In this configuration, SPs are excited via an optical waveguide. Light is coupled into the waveguide modes, and when one of these modes is phase-matched with the SP mode—often achieved using a broadband light source, part of the guided light excites surface plasmons. This interaction produces absorption dips at specific wavelengths in the transmitted spectrum, indicating a resonance.

Table 2 shows the values of sensitivity, resolution, and LOD reported by several Authors that have exploited several kinds of SPR commercial instrumentation over the last 20 years. It can be seen that there has been an improvement in the average sensitivity and LOD values in the most recent period, while the resolution values are more or less comparable to those of instruments published in earlier years.

**Table 2:** Overview of Performances Indicators of SPR Sensors

**Indicator**	**Values range**	**Reference**
Sensitivity	Angular modulation 52.6°/RIU to 193.9°/RIU	^108^,^110^
	Wavelength modulation 1000 nm/RIU to 3223 nm/RIU	^111^,^112^
Resolution	2.24 × 10^–6^ RIU to 8.55 × 10^–6^ RIU	^113^,^114^
LOD	0.74 fg/mL to 93.11 fg/mL	^29^,^115^

It is worth to notice that the values of the resolution reported in Table 2 are worse than those ones reported in the exhaustive work of Homola^116^ in which the best values reached at that time (2009) were reported and compared to the resolution limit theoretically expected. However, those values refer to experimental layouts, for which the translation in operational conditions in the real world operational conditions is usually difficult to implement for several reasons, mainly linked to the difficulty of engineering the setup to a commercial level with reasonable costs.

### 4.5 Comparison with Other Biosensing Techniques

Compared with other label-free techniques that may lack real-time capabilities or have lower sensitivity, SPR is a versatile tool that offers real-time, label-free monitoring of biomolecular interactions. Key advantages include the ability to study kinetics and affinity, handle complex samples, use small sample volumes, and perform high-throughput analysis with techniques like SPRi.

SPR demonstrates several advantages over electrochemical sensors in biosensing applications. While electrochemical sensors are valued for their cost-effectiveness, rapid response, user-friendliness, good sensitivity, low limits of detection, and ease of miniaturization, they typically require labeling of the sample and immobilization on the electrode. Furthermore, these sensors generally target specific redox-active species and cannot provide real-time information on molecular interactions.^117^

Photonic crystals (PCs) represent a significant advancement in medical diagnostics. They are formed by arranging materials with differing refractive indices in one, two, or three dimensions in a periodic structure. This periodicity creates a photonic bandgap, preventing light propagation through the crystal. Introducing structural defects allows selective light transmission within the bandgap.^118^ PC-based biosensors detect changes in the refractive index caused by interactions between the sample and the PC structure. They are appreciated for their compactness, simplicity, low cost, high sensitivity, and label-free operation. PCs sense refractive index changes in the confined optical field, which is influenced by alterations in the composition of biological fluids caused by disease. However, despite their ability to create strong light–matter interactions at small scales, PCs generally exhibit lower sensitivity than SPR because their primary advantage lies in light dispersion rather than direct surface sensing.^119^ Recent developments have focused on integrating SPR with photonic crystals, especially in the form of photonic crystal fibers, to produce hybrid sensors that combine the high sensitivity of SPR with the tunability and enhanced light–matter interaction of PCs for advanced biosensing and chemical detection applications.^119^

Microcantilever biosensing is a technology for detecting analytes that uses the mechanical bending of a microblade (microcantilever) to convert a biological event into a measurable signal. By immobilizing biological recognition elements on the surface of the microcantilever, interactions with the target analyte cause changes in weight or adhesion, inducing a deflection that is detected by a measurement system, often a laser beam. This technique offers the ability to detect specific biological analytes such as DNA, proteins, and antigens, often quickly, sensitively, and without the need for labels.^120^ However, although SPR can analyze complex biological fluids, such as serum, plasma, and cell lysates, without extensive sample purification, microcantilever sensors generally require more purified samples. Moreover, microcantilever sensor chips can be reusable but may have more complex cleaning processes depending on the application. They lack direct kinetic analysis capabilities of SPR, particularly in complex biological matrices, and they do not allow for the simultaneous analysis of multiple analytes on a single chip.^121^

Table 3 summarizes a comparative overview of SPR biosensors and several alternative sensing technologies including electrochemical, photonic crystal, optical fiber, optical waveguide, nanopore, and CRISPR-based sensors. Each technology exhibits unique operational mechanisms, advantages, and inherent limitations that influence its applicability across biomedical, environmental, and diagnostic contexts.

**Table 3:** Comparison of SPR Characteristics versus Competing Biosensing Platforms

**Parameter**	**SPR**	**Electrochemical Sensors**	**Photonic Crystal Sensors**	**Optical Fiber Sensors**
*Detection Principle*	Resonant excitation of surface plasmons at a metal–dielectric interface; refractive index transduction.	Electrical current or potential changes due to redox or charge-transfer reactions.	Optical resonance shift due to refractive index variation in periodic dielectric structures.	Light coupling variations in fiber core/cladding interacting with analyte.
*Typical Detection Limit (LOD)*	5 × 10^–8^ – 5 × 10^–11^ g/mL	5 × 10^–5^ – 5 × 10^–8^ g/mL	5 × 10^–6^ – 5 × 10^–9^ g/mL	5 × 10^–7^ – 5 × 10^–10^ g/mL
*Sensitivity*	Extremely high; allows kinetic quantification of biomolecular binding.	Moderate to high depending on electrode material and surface modification.	Moderate; tunable with photonic bandgap engineering.	High for localized field interaction; depends on mode confinement.
*Sensor Format*	Prism-based, waveguide-coupled, nanoplasmonic chip, or fiber-based designs.	Planar electrodes, microelectrode arrays, microfluidic chips.	Photonic crystal slabs, nanobeams, or microspheres.	Single-mode, tapered, or microstructured optical fibers.
*Label-Free Detection*	Yes, inherently label-free.	Requires redox mediators or enzyme labels in most cases.	Label-free or fluorescently enhanced.	Yes, label-free optical interrogation.
*Real-Time Monitoring*	Yes, continuous and label-free real-time monitoring.	Generally end-point; continuous monitoring less common.	Possible but slower due to optical setup limitations.	Yes, in situ and real-time under flow conditions.
*Multiplexing Capability*	High (microarray or imaging SPR).	Moderate (multielectrode arrays).	High (multiwavelength or spatially resolved).	Moderate (fiber arrays or wavelength division).
*Integration with Microfluidics*	Excellent; supports kinetic assays and automation.	Easy and low-cost integration.	Challenging optical coupling.	Compatible; supports flow sensing.
*Wearable/Portable Potential*	Increasing via fiber-based and flexible plasmonic chips.	Very high; already used in portable devices.	Limited by optical alignment and fragility.	High; flexible optical fibers suitable for wearables.
*Data Processing and AI Integration*	High potential; supports AI-based spectral and kinetic data analysis.	Moderate; AI assists in signal calibration.	Moderate; AI for resonance tracking.	High; supports distributed sensing and demodulation.
*Main Limitations*	Requires precise optical alignment; metal degradation and temperature drift.	Limited selectivity, biofouling, and slower kinetics compared to SPR, which offers real-time, label-free optical detection.	Complex fabrication and temperature sensitivity, making them less robust and scalable compared to SPR platforms.	Fragility and alignment sensitivity limit their stability compared to SPR systems with more stable planar or chip-based configurations.
*Key Applications*	Biomolecular interactions, drug screening, pathogen and biomarker detection.	Glucose, neurotransmitters, ions, environmental toxins.	Protein and nucleic acid detection, optical sensing.	Remote sensing, wearable monitoring, environmental detection.
*Translational Potential*	High—real-time, multiplexed, label-free analysis suitable for clinical translation.	Very high—portable, low-cost, well-established.	Moderate—precision but fragile and complex.	High—miniaturizable and compatible with remote sensing.

**Parameter**	**Optical Waveguide Sensors**	**Nanopore Sensors**	**CRISPR-based Sensors**
*Detection Principle*	Guided optical modes modulated by analyte-induced refractive index change on planar waveguides.	Ion current modulation through a nanoscale pore upon analyte passage or binding.	Cas enzyme-mediated cleavage of reporter molecules triggered by nucleic acid recognition.
*Typical Detection Limit (LOD)*	5 × 10^–8^ – 5 × 10^–11^ g/mL (10^–9^ – 10^–12^ M)	∼5 × 10^–14^ g/mL (single-molecule equivalent)	5 × 10^–14^ – 5 × 10^–17^ g/mL
*Sensitivity*	Very high; strong mode confinement enhances analyte response.	Ultrahigh; based on direct molecular translocation or current blockade.	Very high due to enzymatic amplification (Cas12/Cas13 activity).
*Sensor Format*	Planar integrated photonic chips, Mach–Zehnder interferometers, ring resonators.	Solid-state or biological nanopores embedded in membranes.	Paper-based, microfluidic or chip-integrated CRISPR reaction systems.
*Label-Free Detection*	Yes, refractive index-based and label-free.	Yes, direct detection via current modulation.	Typically requires fluorescent, electrochemical, or colorimetric reporters.
*Real-Time Monitoring*	Yes, capable of high temporal resolution.	Yes, single-molecule, real-time translocation monitoring.	Limited; typically end point reactions with rapid response (<30 min).
*Multiplexing Capability*	High (planar integration allows multiplexing).	Moderate (pore arrays possible).	High via programmable guide RNA arrays.
*Integration with Microfluidics*	Highly compatible; integrated photonics and lab-on-chip.	Moderate; requires precise fluid control for single-molecule analysis.	Excellent; microfluidic lab-on-chip formats widely demonstrated.
*Wearable/Portable Potential*	Moderate to high; integrated photonic chips are miniaturizable.	Low portability due to fluidic and electrical noise control requirements.	High; paper-based and portable CRISPR diagnostic kits emerging.
*Data Processing and AI Integration*	High; AI for mode tracking and spectral interpretation.	Very high; AI for event classification and noise reduction.	High; AI supports automated signal recognition and quantification.
*Main Limitations*	Integration complexity and cost of photonic chips make them less accessible than SPR’s simpler optical setups.	Low throughput, clogging, and signal noise remain major issues compared to SPR’s higher reproducibility and multiplexing capability.	Dependence on enzymatic reagents and end point reactions contrasts with SPR’s real-time, reusable, label-free detection capability.
*Key Applications*	On-chip biosensing, environmental and clinical assays.	Single-molecule DNA/RNA sequencing, viral and protein detection.	Point-of-care nucleic acid testing (SARS-CoV-2, HPV, malaria).
*Translational Potential*	High—strong integration with photonics industry.	Moderate—specialized and infrastructure-dependent.	Very high—low-cost, rapid, user-friendly diagnostics.

## 5 Recent Advances in SPR Biosensor Technology

### 5.1 LSPR and SPRi

The SPR technique has advanced into new instrumental configurations that provide higher resolution and an enhanced signal output.

LSPR is an optical phenomenon that arises from the interaction between incident photons and the conduction electrons of a noble metal nanostructure, leading to collective electron oscillations and absorption in the ultraviolet–visible (UV–vis) range at specific wavelengths.^122^ Unlike conventional SPR, where plasmons propagate along the metal-dielectric interface, in LSPR the plasmons oscillate locally within the nanostructure (Figure 9). The electromagnetic field decay length for propagating surface plasmons is typically around 200 nm,^123^ whereas for localized plasmons it is only a few nanometers,^124^ with both decaying exponentially. This shorter decay length in LSPR reduces interference from bulk refractive index fluctuations while enhancing sensitivity to changes occurring directly at the sensor surface, making it particularly suitable for biosensing applications.^125^

**Figure 9:**
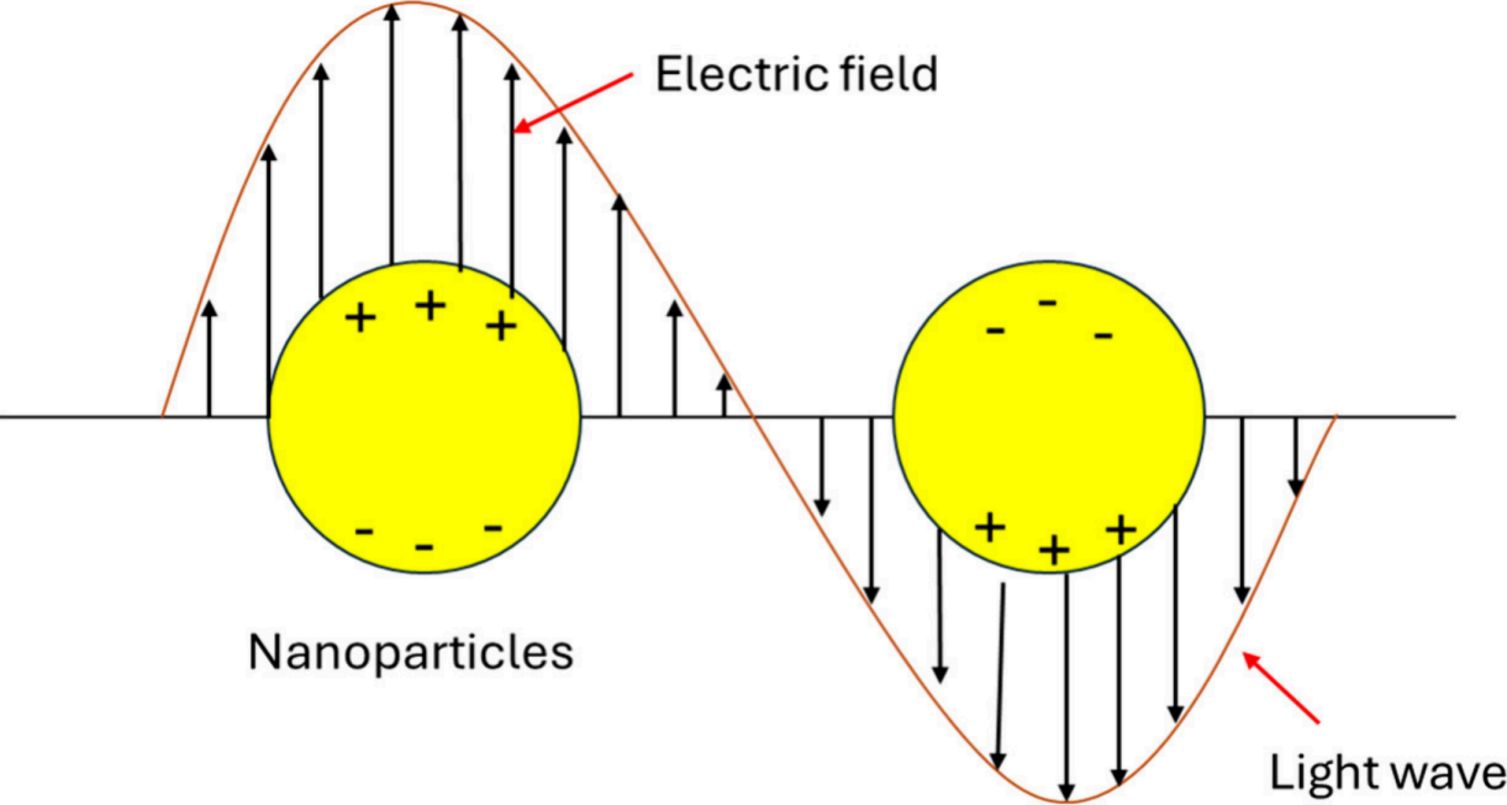
LSPR scheme. The electron clouds oscillate opposite from the direction of the electric field close around the nanoparticles with a size much smaller than the incident wavelength.

LSPR offers several practical advantages over traditional SPR.^126^ The required optical setup is simpler because no prism is needed to couple the light, enabling smaller, more cost-effective instruments. LSPR measurements do not rely on detecting the angle of reflected light, which makes the system more resistant to vibrations and mechanical disturbances. Additionally, the LSPR is less affected by changes in the bulk refractive index, reducing potential measurement errors. Temperature control is less critical, and sensor chips can be manufactured at a lower cost, further simplifying the device production.

SPRi is a sensing approach that leverages evanescent waves for the in situ detection of biochemical samples.^100^ While it operates on the same fundamental principles as conventional SPR, SPRi acquires information about the refractive index changes on the sensor surface through image capture and analysis, typically using a CCD camera for signal detection (Figure 10).^127^ The detection process involves monitoring changes in the image gray scale at a fixed angle of incidence. A key advantage of SPRi is its two-dimensional spatial resolution, which allows simultaneous monitoring of multiple locations on the sensor surface combined with high temporal resolution for real-time tracking of analyte interactions. Unlike spectral SPR, where a single averaged signal is recorded, SPRi detects parallel binding events across a spatially functionalized metal surface. Variations in chemical composition or layer thickness near the metal alter the local dielectric constant, producing a contrast in the recorded images. Binding events can also be visualized by generating difference images, created by subtracting a reference image from a postbinding image. This imaging capability enables researchers to evaluate and compare different surface modifications under identical experimental conditions, whether by analyzing the same ligand with multiple receptor types or by assessing the effects of various chemical treatments. SPRi therefore facilitates homogeneous data acquisition and accelerates analysis. Its spatially resolved measurements make it particularly well-suited for examining complex samples, such as cell arrays, whole-cell interactions, and large biomolecule arrays, providing detailed insight into local binding events rather than a bulk-averaged response.^128^

**Figure 10:**
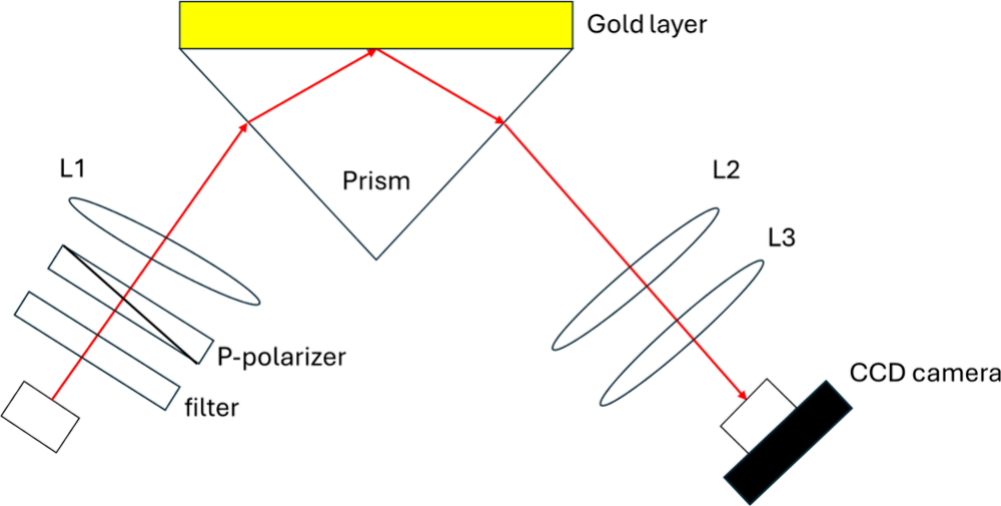
SPRi Setup: In this configuration, the light first passes through collimating lenses and then through a narrow-band interference filter and a polarizer. This produces a monochromatic and polarized beam that is directed onto the prism coupler. The light reflected from the gold-coated sensor surface is captured by a monochromatic CCD camera. Additional lenses (L2 and L3) are positioned in front of the CCD to enhance image quality. The captured images can be digitally stored and subsequently processed using image analysis software for a detailed evaluation.

### 5.2 Latest Technological Developments

In recent years, SPR systems integrated with complementary techniques have been developed to enhance the sensitivity and improve the accuracy of detecting small amounts of analytes.

Electrochemiluminescence sensors rely on redox luminescence phenomena, triggered by radiative charge reactions between positive and negative radicals generated on the electrode surface after electrochemical stimulation. The resulting luminescence intensity is highly sensitive to the concentration of surrounding biomarkers.^100^ Electrochemiluminescence sensors offer advantages, such as low cost, broad dynamic range, high sensitivity, and operational simplicity. However, challenges remain including complex electrode modification, susceptibility to interference from other currents, limitations in sample volume, the requirement of markers for molecules lacking direct electron transfer, and difficulty in dynamically tracking biomolecular interactions on the sensor surface.

SPR Microscopy (SPRM) was developed to overcome the low spatial resolution of conventional SPRi, which cannot detect single molecules or nanoparticles. Two main SPRM configurations exist: prism-based and objective-based. Prism-based SPRM offers higher sensitivity and throughput but is limited by objective lens working distance and imaging distortion, making submicrometer molecular dynamics difficult to resolve. Objective-based SPRM, with higher spatial resolution, is better suited to studying sparse particles. Total internal reflection-based SPRM (TIR-SPRM) further enhances the resolution by using a wavelength-tunable femtosecond laser, which provides excellent beam parallelism and broad spectral coverage to minimize speckle noise. A 3D displacement stage allows simultaneous optimization of angle and wavelength to locate maximum absorption without prior knowledge of the sample’s refractive index. Using this system, specific binding events—such as human IgG and goat antihuman IgG antibodies—can be monitored with imaging resolution down to 248 nm, with nanoparticle image distortion minimized via ring filters and Fourier-domain deconvolution algorithms.^129^

Hyperspectral SPR Microscopy (HSPRM) employs a hyperspectral microscope to analyze selected areas of SPR images generated by prism-based spectral SPR sensors.^130^ Hyperspectral imaging provides both high spectral and spatial resolution, generating a three-dimensional data cube capturing the spectral information on each pixel. HSPRM enables monochromatic and polychromatic SPRi, single-pixel spectral SPR sensing, and two-dimensional quantification of thin films using measured resonance wavelengths. Its features include a wide spectral range (400–1000 nm), flexible field of view (0.884–0.003 mm^2^), and high lateral resolution (1.2 μm), representing a significant advancement in SPR sensor technology.

Multiparametric SPR (MP-SPR) spectroscopy allows real-time monitoring of molecule immobilization on the sensor surface, followed by characterization of concentration-dependent activity.^131^ Unlike traditional SPR, MP-SPR uses multiple wavelengths, permitting not only kinetic analysis of biomolecular interactions but also direct surface characterization, including layer thickness (up to several micrometers), refractive index, and surface coverage. Advanced goniometric configurations enable a broad angular scanning range (≈40°–78°), multiple wavelengths, and automated fluidics. This system continuously records the entire biosensing process, provides direct and precise layer thickness measurements by modeling SPR curves at various wavelengths, and allows for simultaneous monitoring of additional optical parameters without prior assumptions. MP-SPR is label-free, does not require electroactive species, and minimizes interference, ensuring that surface measurements accurately reflect molecular interactions.

Recent advances in micro- and nanofabrication have facilitated the development of nanostructured SPR substrates, including metallic nanohole, nanoring, and nanomushroom arrays^132^ (Figure 11). Compared with conventional prism-based SPR sensors or plasmonic nanoparticles, these nanostructured sensors can be directly integrated with imaging and microfluidic systems. They allow plasmonic resonances to be excited without additional coupling structures, enabling high-throughput, multiplexed analyses and facilitating miniaturization and portability. The versatile nanostructure designs also enhance near-field intensities, reduce resonance line widths, and improve the figure of merit (FOM), making them highly suitable for medical diagnostics, food safety, environmental monitoring, and more. For example, Alkorbi et al.^133^ developed a highly sensitive metasurface SPR biosensor combining graphene and gold nanostructures with circular, ring, and rectangular resonator elements. Through numerical simulation and optimization, the geometrical parameters were fine-tuned to maximize performance, achieving a sensitivity of 200 GHz/RIU and a detection limit of 0.10285 RIU across a refractive index range of 1.33–1.4. This high sensitivity enables precise detection of minute changes in the analyte concentration, highlighting the potential of nanostructured SPR platforms for advanced biosensing applications.

**Figure 11:**
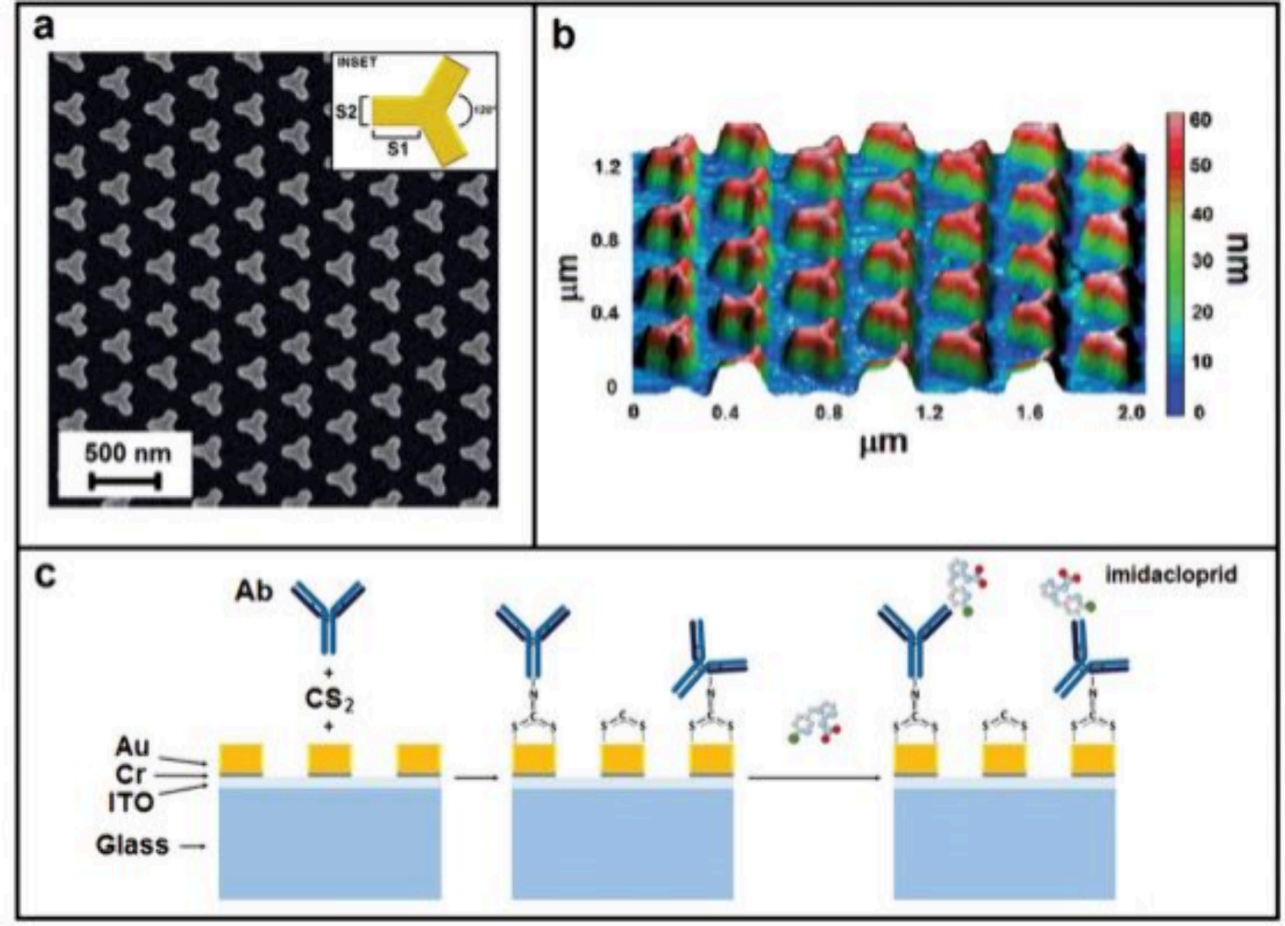
Scheme of an example of a nanostructured SPR sensor morphology.^134^ a) Image of the nanostructures with SEM. b) image of the nanostructures with AFM. c) Sketch of the multilayer and illustration of the steps of functionalization.

Microfluidic systems for high-throughput SPR detection must accurately and rapidly deliver analytes and reagents while ensuring uniform flow across the sensor surface.^135^ Microfluidics refers to the manipulation and control of fluids on a submillimeter scale, typically within channels smaller than one millimeter.^136^ These systems can automate chemical analysis by integrating essential steps such as sample transport, chemical reactions, and detection. Microfluidics also reduces sample and reagent consumption to the nanoliter scale and accelerates the mixing of reagents, which enhances the performance of SPR biosensors. Integration of microfluidic chips with SPR systems enables the ultrasensitive, multiplexed detection of analytes, allowing simultaneous monitoring of multiple biomarkers. However, key challenges remain, including surface fouling that can decrease sensor sensitivity, reproducibility of microchannel fabrication, and integration of optical components without compromising the SPR resonance quality. Careful design is also required to prevent sample cross-contamination and maintain laminar flow for accurate kinetic measurements.^136^ To address these challenges in point-of-care (POC) testing, Fan et al.^137^ developed a smartphone-based LSPR biosensor system with an integrated multichannel microfluidic platform, termed SBSM (Smartphone-Based Multitesting). The SBSM incorporates nine sensor units capable of simultaneous detection of multiple biomarkers, with 72 additional 72 sensor units fabricated for validation. Its modular design includes a light source, lenses, a grating, a case, and a smartphone shell, which can be easily assembled and attached to a smartphone. Performance testing of the SBSM demonstrated a sensitivity of 161.0 nm/RIU and a limit of detection (LOD) of 4.2 U/mL for CA125 and 0.87 U/mL for CA15–3 in clinical serum specimens. The results were highly correlated with those of conventional ELISA assays, confirming the reliability and accuracy of the system. Furthermore, the SBSM is user-friendly and requires minimal professional training. Due to its compact size, multitesting capability, and customizable design, the SBSM represents a promising platform for POC detection of multiple biomarkers.

Hybrid plasmonic–photonic SPR systems have emerged as a cutting-edge research direction at the interface between nanophotonics and plasmonics, aimed at achieving unprecedented control over light–matter interactions at the nanoscale. Traditional plasmonic sensors exploit the excitation of surface plasmon polaritons or LSPR at metal–dielectric interfaces, providing extreme field confinement and high RI sensitivity. However, their performance is fundamentally limited by ohmic losses in metals, which reduce the propagation length and degrade the quality factor of the optical resonance. Conversely, purely photonic structures, such as dielectric waveguides or photonic crystal cavities, offer low-loss light guiding and high-quality factor resonances but suffer from weak field confinement due to the diffraction limit. By combining these two regimes into hybrid plasmonic–photonic architectures, researchers aim to overcome the intrinsic limitations of each platform, realizing devices that simultaneously exhibit strong field localization, enhanced sensitivity, and low propagation loss. Such systems have shown particular promise for label-free biosensing, environmental monitoring, on-chip spectroscopy, and integrated optoelectronics, where compactness, tunability, and compatibility with complementary metal-oxide-semiconductor fabrication are critical.^138^ An example is the bimodal plasmonic RI sensors based on SU-8 waveguides, in which an aluminum stripe combined with a bilayer SU-8 photonic waveguide core and polymer cladding achieved a sensitivity of approximately 6300 ± 460 nm/RIU, surpassing many traditional and polymer-based plasmophotonic sensors.^139^

## 6 Recent Advances in SPR Biosensor Design

Magnetic nanoparticles (MNPs) provide an effective solution to improve sensitivity and reduce nonspecific adsorption in the preconcentration and isolation of target analytes.^140^ In recent years, MNP-based sensing strategies have been developed for ultrasensitive detection of cells, nucleic acids, proteins, and small biomolecules. Typically, MNPs are functionalized with specific receptors to capture target analytes, which are then collected by using a magnetic field. The analyte-bound MNPs can either be brought to the sensor surface or redispersed in solution after extraction. For example, electrochemical magnetobiosensors collect analyte-bound MNPs on magnetic electrodes, generating strong electrochemical signals. Alternatively, MNPs can be captured on nucleic-acid- or antibody-modified surfaces to produce optical signals. Notably, MNPs enhance SPR signals due to their high molecular mass, elevated refractive index, low production cost, and ease of synthesis via hydrothermal or coprecipitation methods. Despite these advantages, challenges remain for real-time SPR assays using MNPs.^141^ For instance, sensor chips must be functionalized with specific receptors under controlled conditions, and capturing analyte-bound MNPs often requires long hybridization times and low flow rates. Additionally, receptor immobilization on the chip can create steric hindrance, limiting the efficient capture at the solid–liquid interface. Consequently, there is a need for novel MNP-based SPR designs that simplify detection, reduce analysis time, and improve the efficiency.

Graphene and its derivatives are commonly combined with metals to induce larger SPR signal changes than bare metal films.^142^ When incorporated into composites, graphene enhances sensitivity by intensifying the local electromagnetic field at the sensor interface. Its optical properties shift SPR curves and amplify the refractive index response. Graphene can be coupled with plasmonic metals, such as gold or silver, enabling efficient use in biomedical and clinical diagnostics. Functionalization allows graphene to serve as a scaffold for enzyme immobilization or molecular doping, maximizing the level of biomolecule extraction per unit area. Graphene-based SPR sensors have gained attention due to their enhanced sensitivity, selectivity, and functionalization potential. For instance, Tene et al.^143^ designed a multilayer SPR sensor integrating silver, silicon nitride, single-layer graphene, and thiol-tethered ssDNA for SARS-CoV-2 detection. This system achieved a sensitivity of ∼315.91°/RIU, high stability, linearity, and low detection limits, highlighting graphene’s dual role in improving both biorecognition and optical field interaction. Research continues on integrating graphene-enhanced SPR with flexible substrates, microfluidics, and photonic circuits for portable, label-free, and point-of-care diagnostics. Limitations include the challenge of detecting very small analytes and interference from bulk refractive index changes or inherent optical properties of the sample, which can reduce measurement accuracy.^144^

Molybdenum disulfide (MoS_2_) is another 2D material increasingly used in SPR applications.^44^ MoS_2_ has higher optical absorption than graphene, exceptional optical and electrical properties, and low cytotoxicity, making it suitable for biosensing. Its large surface area and free sulfur atoms facilitate the development of biosensor interface development. When deposited on metal films, MoS_2_ layers enable strong coupling at the metal/MoS_2_ interface via charge transfer and field enhancement, increasing SPR sensitivity. However, achieving uniform monolayer distribution over large areas remains a significant challenge for MoS_2_-based SPR sensors.^145^

## 7 Machine Learning in SPR Biosensors

Smart devices integrating sensors with artificial intelligence (AI) are increasingly prevalent in industrial, healthcare, and home environments. Advances in AI and machine learning (ML), combined with large-scale data storage and cloud computing, have enabled consumer devices, such as Alexa, Siri, and Google Home. Traditional ML approaches typically rely on selecting specific features to achieve desired outcomes.^146^ Modern intelligent sensors enhanced with ML are capable of automated monitoring, predictive maintenance, and fault detection. ML algorithms can also integrate sensor data with computer simulations to predict the behavior of complex systems. SciML, for instance, allows the creation of virtual sensors to estimate parameters that cannot be directly measured by leveraging data from existing functional sensors.^147^ AI-based data processing enables extraction of meaningful information from noisy or low-resolution sensor outputs, revealing correlations between analyte properties and sensor signals as well as identifying anomalies due to biofouling or other interferences.

ML has shown particular promise in enhancing the performance and cost-effectiveness of SPR sensors. During angle- or wavelength-interrogation measurements, ML algorithms can process large volumes of reflectance data, improving the signal extraction from noisy sources. This capability enables the use of lower-power light sources, reducing both cost and safety concerns. Environmental factors, such as temperature, humidity, and pressure, can induce cross-sensitivity in SPR sensors. ML approaches can account for these variables, enabling multiparameter sensing that compensates for environmental fluctuations. Furthermore, the dynamic responses of SPR sensors often exhibit nonlinearities, including drift and short-term transients. ML algorithms can distinguish meaningful signal components from such effects, which improves measurement accuracy. Applications of ML in SPR include optimizing plasmonic coatings and processing sensorgrams. For example, genetic algorithm-based neural networks have been employed to guide the seed-mediated growth of sea urchin-like gold nanoparticles to achieve specific plasmonic wavelengths.^148^ In another study, a surface plasmon resonance imaging (SPRi) system combined with carbohydrate microarrays was used to detect multiple sclerosis biomarkers in undiluted whole serum. ML algorithms analyzed the SPRi data, accounting for cross-reactivity between antibodies, and evaluated both kinetic and steady-state binding components. This approach enabled highly sensitive detection, achieving a limit of detection below 7 ng/mL for analyte concentrations in the 1–100 ng/mL range.^149^

Conventional SPR data analysis often relies on simplified kinetic models (e.g., Langmuir), which fail to capture nonlinearities present in real biological samples. ML approaches, including convolutional and recurrent neural networks, have been successfully applied to denoise sensograms, automatically extract kinetic parameters, and classify binding behaviors in complex fluids.^150^ These methods outperform traditional curve-fitting by learning the intrinsic relationships between response patterns and kinetic constants, achieving up to 70% reduction in variance for dissociation constant determination under noisy conditions. Furthermore, pattern recognition models trained on SPRi data sets have demonstrated high diagnostic performance, distinguishing pathological from healthy samples with >94% sensitivity and >96% specificity in oncological plasma profiling.^151^ In addition, adaptive ML algorithms can dynamically correct baseline drift and fouling effects during repeated sensor regeneration cycles, extending chip lifetime and improving reproducibility in clinical environments.^152^ Integrating these artificial intelligence-driven analytical layers into next-generation PoC SPR platforms allows automated, real-time interpretation of biosensing data, reducing operator dependency, and enhancing diagnostic robustness.

Despite its potential to improve the performance of SPR biosensors, machine learning in sensor design still faces several challenges. Very few publicly available data sets can be used to train the ML algorithms, which also require improvements in SPR sensor design for standardization and a global framework for ML-based SPR biosensors. The widespread application of data-driven SPR requires the development of robust algorithms. There is also a need to ensure algorithm interpretability and generalizability across different sensor platforms and biological matrices.^146^

## 8 Future Challenges

### 8.1 POC Systems

SPR technology still has wide margins for improvement and is taking on an increasingly important role in the market for devices with potential medical applications. Its versatility is key to pursuing new strategies and offering new solutions. Certainly, one viable path is toward POC systems. SPR sensors are being integrated into small, portable devices to facilitate analysis outside of specialized laboratories and at the patient’s bedside.^153^ This would allow for the detection of disease-specific molecules in biological fluids, such as saliva and blood, to enable the early diagnosis of conditions such as periodontitis and inflammatory diseases. It would also permit real-time monitoring of biomarkers, especially during disease outbreaks or chronic conditions. Furthermore, it would leverage wireless features and contribute to personalized medicine by providing precise and accessible diagnostics directly to patients, thereby improving treatment decisions. Moreover, multiplexed sensing capabilities are essential for simultaneously detecting multiple biomarkers and providing a comprehensive health profile of the patient.

The future clinical viability of SPR biosensors increasingly depends on advances in miniaturization, optical portability, and microfluidic integration. Traditional SPR systems are instruments requiring precise optical alignment, stable environmental conditions, and the manual handling of reagents, limiting their applicability in decentralized settings. Recent technological developments aim to reduce the footprint of SPR platforms through compact optics, portable light sources such as LEDs or miniaturized lasers, and simplified detection schemes that preserve sensitivity while enhancing robustness.^51^ In parallel, microfluidic integration allows precise control over sample delivery, reaction timing, and multiplexed analysis, while minimizing sample volume and enabling automation of preanalytical processes, such as plasma separation or target enrichment.^154^

### 8.2 Toward Fiber-Optic Structure

Among the main plasmonic coupling configurations used in SPR biosensors, fiber-optic architecture is emerging as the most promising platform for future portable PoC applications. While the prism-based configuration provides excellent sensitivity and angular resolution, it requires complex and bulky optical alignment systems that limit its miniaturization and field deployment. Grating-coupled SPR offers a more compact planar geometry compatible with lab-on-chip integration; however, it still depends on precise nanofabrication and is sensitive to angular and spectral instabilities. In contrast, fiber-optic SPR sensors^155^ provide intrinsic optical alignment, extreme miniaturization, and seamless integration with low-cost light sources and miniaturized spectrometers, enabling portable and cost-effective operation.^156^ These systems exhibit high mechanical robustness, can be directly coupled with disposable microfluidic cartridges, and allow facile surface functionalization of the metallic coating (Au or Ag) for selective biomolecular recognition. Geometrical variants such as D-shaped, tapered, or U-bent fibers enhance the interaction of the evanescent field with the surrounding medium, achieving sensitivities comparable to traditional prism systems.^157^ Recently, hybrid designs combining fiber-optic SPR with LSPR have further improved thermal stability and detection sensitivity, paving the way for fully integrated PoC diagnostic devices.^158^ Consequently, the fiber-optic coupling configuration is considered to be the most likely to dominate the next generation of portable SPR biosensors, providing the optimal balance between sensitivity, robustness, and integrability for clinical and field-based diagnostics.

### 8.3 Wearable Sensors and Cloud-Based Diagnostics

In recent years, flexible and biocompatible substrates are increasingly being explored to integrate SPR sensors into wearable formats, enabling continuous, noninvasive, and real-time monitoring of biomarkers in biofluids such as sweat, saliva, or interstitial fluid.^159^ Such wearable platforms have the potential to provide dynamic health information for personalized medicine, including real-time tracking of metabolites, stress markers, immune responses, or hormone fluctuations. Recent advances involve the use of stretchable plasmonic nanostructures, transparent conductive electrodes, and protective coatings that maintain the sensor performance under mechanical deformation, bending, or stretching. Furthermore, integration with miniaturized optics, microfluidic channels for localized sample collection, and wireless electronics facilitates on-body operation without hindering mobility. Despite these advances, key challenges remain: ensuring long-term chemical and mechanical stability, achieving uniform and reproducible optical coupling under motion or skin deformation, and designing biocompatible and antifouling surface functionalizations that preserve selectivity over extended use. Additionally, miniaturization must be balanced with maintaining sufficient plasmonic field intensity to achieve high sensitivity, and integration with low-power electronics is essential for continuous monitoring in real-world applications.

The integration of SPR sensors with cloud computing infrastructures opens new possibilities for remote, large-scale, and intelligent diagnostic applications. By streaming sensor data to cloud platforms, real-time analysis, predictive modeling, longitudinal monitoring, and population-level epidemiological studies. Cloud-based architectures also enable integration with electronic health records, telemedicine frameworks, and decision-support systems, providing clinicians with actionable insights from distributed SPR networks. Key technical challenges include ensuring data security and patient privacy, establishing reliable high-speed connectivity across diverse environments, and standardizing data formats and metadata to allow for interoperability between different sensor platforms. Moreover, the real-time processing of streaming data requires the development of robust, scalable algorithms capable of handling large volumes of heterogeneous information without latency or loss of fidelity. The combination of wearable SPR devices with cloud-based analytics has the potential to create an ecosystem for continuous, personalized, and predictive healthcare, but its success will depend on addressing these infrastructural, computational, and regulatory challenges.^160^

### 8.4 Market Growth

The status of the annual publication with the keyword “surface plasmon resonance” search on the PubMed database over the past five years is shown in Figure 12.

**Figure 12:**
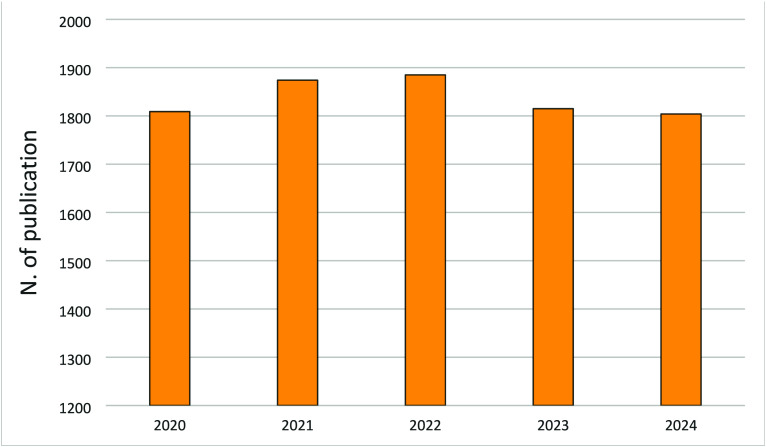
Publications status in each year on PubMed.

The chart above illustrates trends in interest and investment in SPR research over time. Although there has been a slight decline in the past two years, it will be interesting to monitor how these trends evolve and what new discoveries may emerge. The bar diagram highlights sustained and significant investment in SPR, reflecting its crucial role in advancing scientific research and technological innovation. According to a report by Verified Market Research, the global SPR market was valued at approximately USD 725 million in 2023 and is expected to surpass USD 1.247 billion by 2030, corresponding to a compound annual growth rate (CAGR) of 9.12% between 2024 and 2030.^161^ Similarly, Future Market Insights forecasts that the SPR market will grow from USD 1.0 billion in 2024 to USD 1.6 billion by 2034, while Coherent Market Insights projects that it will reach USD 1.5 billion by 2030. These estimates indicate consistent year-on-year growth, driven by rising market demand, declining production costs, and the performance advantages of SPR technologies, supporting their strong commercial potential.

## 9 Conclusion

Over the past years, SPR biosensors have demonstrated remarkable versatility in detecting a wide range of chemical and biological analytes, including tumor biomarkers, pathogens, and rare disease indicators. The continuous improvement in sensitivity, precision, and real-time detection, along with the reduction of operational steps, has positioned SPR as one of the most promising platforms in biomedical diagnostics. Recent advances in nanostructuring, plasmonic hybridization, and surface chemistry have enhanced analyte binding efficiency and minimized nonspecific interactions, strengthening the translational potential of this technology.

Despite these achievements, several challenges remain before SPR biosensors can be fully established as clinical and POC diagnostic tools. One major issue lies in the reproducibility of surface functionalization, as minor variations in immobilization chemistry can lead to significant discrepancies in sensor performance. Standardization of surface modification protocols, automated microfabrication, and the use of robust SAMs or bioinspired coatings could improve interdevice consistency and long-term stability.

Another critical challenge concerns biofouling, particularly in complex biological samples, such as serum or whole blood. The adsorption of nonspecific proteins or lipids on the metal surface often compromises sensitivity and reproducibility. Promising mitigation strategies include the development of antifouling polymer brushes, zwitterionic interfaces, and nanostructured hydrophilic coatings capable of preserving signal integrity during prolonged use.

From a translational perspective, clinical validation remains a key bottleneck. Although numerous laboratory studies have demonstrated impressive analytical performance, large-scale comparative trials against gold-standard diagnostic assays are still limited. The implementation of standardized validation frameworks, in collaboration with clinical laboratories and regulatory bodies, is essential to ensure reliability, safety, and reproducibility under real-world conditions.

Looking forward, the path toward POC SPR diagnostics involves convergence with microfluidics, wearable technologies, and AI-driven data analysis. The miniaturization of SPR chips, integration with low-power optical components, and wireless data transmission could enable portable, cloud-connected biosensing devices capable of delivering rapid, quantitative results directly at the patient’s bedside. Coupling SPR data with ML algorithms for pattern recognition and anomaly detection will further enhance the analytical accuracy and facilitate automated decision support in personalized medicine.

In summary, while SPR biosensing has matured into a powerful analytical technology, its future clinical impact will depend on overcoming the current limitations in surface reproducibility, antifouling performance, and clinical validation. Through standardization, smart materials engineering, and digital integration, SPR systems are poised to transition from laboratory tools to next-generation POC diagnostics capable of transforming clinical workflows and precision medicine.
